# From microstates to macroscales: A Critical Review of Maximum Entropy Modeling and Energy Landscape Analysis in functional MRI

**DOI:** 10.21203/rs.3.rs-8428652/v1

**Published:** 2025-12-29

**Authors:** Konasale Prasad, Ouyang Bowei, Nicholas Theis

**Affiliations:** 1 Department of Psychiatry, University of Pittsburgh School of Medicine, University of Pittsburgh, Pittsburgh, PA; 2 Department of Bioengineering, Swanson School of Engineering, University of Pittsburgh, Pittsburgh, PA; 3 VA Pittsburgh Healthcare System, Pittsburgh, PA

**Keywords:** Functional MRI, Network Neuroscience, Maximum Entropy Model, Energy landscape, Dynamic functional network, Dynamical system

## Abstract

Functional magnetic resonance imaging has revolutionized neuroscience. Traditional analyses focusing on differences in regional activations and pairwise regional interactions (functional connectivity) cannot capture the collective nature of inherently time-variant network dynamics, which would be crucial to better understand the brain function at the systems level. This systematic review on maximum entropy models (MEMs), derived from statistical physics, critically appraises a principled framework that integrates regional activations and pairwise interactions to characterize patterns of dynamic functional network reorganizations at much shorter timescales. This method provides a global statistical structure of network configurations as energy landscapes enabling the tracking of evolution of network configurations over the duration of fMRI acquisition. Unlike correlation-based functional connectivity that assumes independence of regional correlations, MEMs capture the interdependent nature of network dynamics providing a statistically more persuasive picture of the network. The MEM utilizes binarized activation patterns to estimate the probability of network configurations, assigning “energy” values that represent the statistical likelihood of occurrence of specific brain states. MEMs reveal fundamentally altered dynamic functional network reconfigurations and energy landscapes in schizophrenia and other disorders where patients spend more time in high-energy (low-probability network configuration) brain states associated with cognitive dysfunction and more severe psychopathology. MEM also revealed distinct findings related to autism spectrum disorder, sleep, perception, and memory and show superior correspondence to structural connectivity compared to traditional methods, providing biologically grounded functional biomarkers. This approach bridges statistical physics with systems neuroscience, offering new perspectives on brain criticality and psychiatric pathophysiology.

## 1. Introduction

The human brain is an extraordinarily intricate organ, composed of approximately 90 billion neurons, each capable of forming an estimated 1,000 synaptic connections (Azevedo et al., 2009). This immense network results in trillions of synapses that facilitate a vast array of interactions, making the brain one of the most complex systems known in nature. These neurons engage in continuous communication through electrical impulses and chemical signaling, forming dynamic circuits that underpin all aspects of cognition and behavior. This neural network is not static; rather, it is characterized by dynamic and ever-changing patterns of activity that arise from both stochastic (random) neuronal firing and highly coordinated signaling mechanisms that span multiple spatial and temporal scales—from localized microcircuits to large-scale networks working in synchrony (Shi et al., 2019). Such variability and adaptability in neural activity are essential for the brain’s ability to respond to internal states and external stimuli. Recent research has increasingly highlighted the importance of these dynamic connectivity patterns in supporting core neurocognitive functions. Processes such as perception, memory, reasoning, and decision-making are now understood to emerge not merely from isolated brain regions, but from the fluid and context-dependent interactions among distributed neural networks. Rather than being static or uniform, brain connectivity evolves moment-to-moment, reflecting the brain’s ongoing efforts to integrate sensory inputs, internal goals, and external demands. This perspective underscores the brain’s capacity for flexible and adaptive cognition, driven by the continuous reconfiguration of its functional architecture. Understanding these patterns is essential for unraveling the mechanisms of healthy brain function and for identifying disruptions that may underlie neuropsychiatric disorders.

Understanding neural dynamics increasingly relies on non-invasive methods such as functional magnetic resonance imaging (fMRI). Recent advances in MRI hardware—higher magnetic field strengths, improved gradient coil architectures, and optimized head coil designs (Santini et al., 2021)—are pushing spatial and temporal resolution boundaries. The NextGen 7 Tesla MRI scanner can achieve up to a 20-fold improvement in spatial resolution compared to traditional 7 Tesla systems (Calabro et al., 2024; Feinberg et al., 2023), with acquisitions up to 4–5 times faster than conventional MRI systems (Hendriks et al., 2020). fMRI leverages the blood oxygenation level-dependent (BOLD) signal to infer brain activity. These improvements allow for more precise mapping of neural activity and finer-grained analysis of brain function.

The BOLD signal reflects changes in cerebral blood flow and oxygenation mediated by glia-dependent neurovascular coupling (Schmithorst et al., 2015), maintained through complex signaling pathways initiated by glutamate release from activated neurons (Iadecola, 2017; Schaefer and Iadecola, 2021) along with physiological noise, motion artifacts, and signal dropout (Hoge, 2012). The signals may also be affected by underlying structural alterations (Haszto et al., 2020; Prasad et al., 2016; Prasad et al., 2018). Thus, BOLD signals are complex requiring increasingly sophisticated methods to understand the neural dynamics; for example, the analytical methods should be able to factor in inherent variabilities such as positive BOLD response for excitatory neuronal activity whereas biphasic BOLD signals for inhibitory neurons (Moon et al., 2021) which the traditional methods fail to do.

Since its introduction (Kwong et al., 1992), fMRI has led to diverse analytical methods. Early analyses focused on regional task-related activations using statistical frameworks like the general linear model (Friston et al., 1994a). Functional connectivity (FC) emerged to measure temporal co-activity between spatially distinct brain regions (Biswal et al., 1995). However, both these approaches are limited in leveraging the richness of the BOLD signals. Methods rooted in statistical physics such as the maximum entropy model (MEM) is promising in providing a better understanding of the neural dynamics that is the focus of this review.

## 2. Commonly Used Approaches and Challenges

### 2.1. Groupwise Comparison and Statistical Analysis

**Figure 1:**
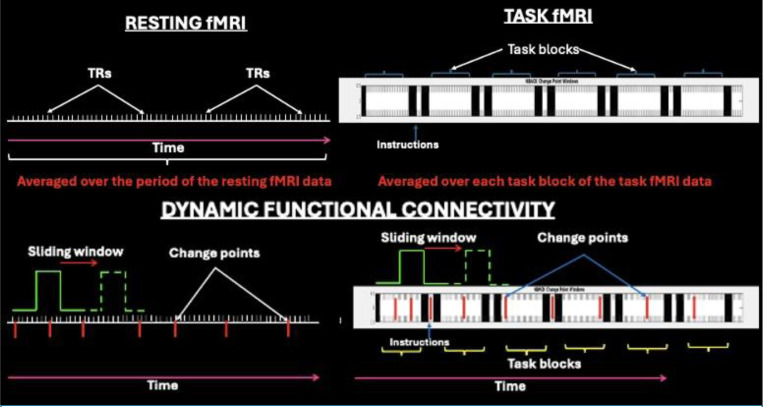
Traditional approaches to investigate resting state fMRI and task-fMRI. Both approaches involve averaging the BOLD response over selected periods of times (Top). Dynamic functional connectivity (bottom). Two major approaches to examine dynamic functional connectivity are shown for the resting state (left) and task (right) fMRI. Sliding windows method uses a window of specified length moved along the time-series data at specified intervals and the connectivity patterns along with measures such as dwell time and frequency of occurrence are estimated. A more advanced method calculates change points using various statistical and mathematical approaches to determine a point where “abrupt” change in network architecture is detected obviating the need for placing and shifting the windows.

Two widely employed methodologies for investigating functional brain activity using fMRI involve the quantification of regional hemodynamic responses under resting-state (not related to explicit engagement in a task) and task-based (engaging and responding to stimuli in a cognitive task) conditions, often called first-order models. The resting-state paradigm acquires (Figure 1, Top Left), BOLD signals over a defined period of data acquisition in the absence of explicit task stimuli or expected behavioral outputs. In contrast, task-based fMRI (Figure 1, Top Right) involves the aggregation of BOLD responses across multiple repetition times (TRs) within a block design framework requiring averaging of BOLD responses, thereby enhancing signal-to-noise ratio (SNR). The TRs generally vary from 1s to 3s in most studies, depending on experimental design and scanner hardware. Such averaging in resting and task fMRI facilitates the identification of generalizable patterns of average brain activation levels associated with cognitive task performance. However, averaging obscures temporal fluctuations that may be important for cognitive processing (Buckner et al., 1998; Dale and Buckner, 1997) limiting the ability to isolate stimulus-specific neural responses. For instance, two individuals or groups may show statistically non-significant differences in average BOLD responses but one may exhibit a stable brain state during the entire duration of averaging while another may be transitioning across different states within short time periods during the acquisition time that is lost when the signals are averaged. Such loss of temporal specificity for variations in BOLD responses and individual variability may constrain the interpretation of findings and limit the clinical applicability and translational potential of these findings. However, several important findings have been reported by comparing BOLD response differences (D’Aiuto et al., 2015; Keshavan et al., 2017; Ragland et al., 2009; Stolz et al., 2012).

Event-related fMRI allows researchers to examine the brain’s response to specific, time-locked stimuli, providing improved temporal resolution compared to traditional block designs. In this approach, stimuli are presented in discrete events, and the resulting BOLD signal is measured in close temporal proximity to each stimulus, enabling the detection of rapid neural responses associated with cognitive processes such as attention, memory encoding, and decision-making. This granular view of brain activity makes it possible to disentangle overlapping neural events and better attribute observed changes in BOLD signal to particular cognitive functions.

Despite these advantages, event-related fMRI faces several challenges. The signal strength is often reduced because the analysis relies on averaging BOLD responses over a long period of time and many events, which can dilute the signal of interest, especially when responses are variable across trials or subjects. Additionally, inter-subject variability in responding to the stimuli can further obscure meaningful patterns and complicate group-level comparisons. These factors necessitate the use of advanced statistical techniques to accurately model and interpret the data to isolate stimulus-locked neural responses by accounting for various confounding factors and temporal dynamics (Poldrack and Farah, 2015). Independent component analysis (ICA) is often applied to decompose the complex BOLD signals into distinct spatial and temporal components, facilitating the identification of functional networks engaged during event-related tasks.

Interpreting hemodynamic changes related to regional BOLD response differences remains challenging. Elevated BOLD responses may reflect increased neural engagement or “inefficient processing” when associated with poorer performance (Callicott et al., 1999) which is often difficult to empirically establish. These analytical approaches have been instrumental in revealing alterations in neural coordination associated with neuropsychiatric disorders. For example, studies using event-related fMRI combined with dynamic connectivity analyses have uncovered disrupted patterns of brain network organization in conditions such as schizophrenia (Ragland et al., 2004), bipolar disorder (Hassel et al., 2009), and major depressive disorder (Wagner et al., 2006). Such findings highlight the clinical relevance of event-related fMRI and underscore the importance of employing robust statistical methods to fully leverage its potential in elucidating the neural mechanisms underlying cognitive dysfunction and psychopathology.

### 2.2 Static Functional Connectivity (sFC)

Correlated BOLD responses between different brain regions—often called second-order models— are used to construct FC networks in both resting-state and task-based fMRI studies. These FC networks reflect correlated BOLD responses that may reflect synchronized activity and possible connection through white matter fibers. Thus, FC allows one to understand how different areas of the brain communicate and coordinate their activity. The construction of FC mirrors the construction of structural covariance networks that show how anatomical features covary across regions (Prasad et al., 2022a; Prasad et al., 2022b). However, FC does not assume a white matter fiber directly connecting the regions that show correlated BOLD responses but may involve higher order white matter connections.

Resting-state functional connectivity (rsFC) refers to measuring how spontaneous BOLD signal fluctuations in spatially distinct brain regions are temporally correlated when a person is not performing any explicit task. Typically, these correlations are averaged over the entire duration of the scan, which helps to identify well-established brain networks known as resting-state networks (RSNs) such as the default mode network (DMN), central executive network (CEN), the frontotemporal network (FPN), the dorsal attention network (DAN), and the salience network (SN).

The DMN is the most extensively studied RSN and includes core regions such as the medial prefrontal cortex, posterior cingulate cortex or retrosplenial cortex, and both left and right inferior parietal lobules (Whitfield-Gabrieli and Ford, 2012). Some studies also include additional regions like hippocampal formation (Buckner et al., 2008) and the thalamus (Menon, 2023) as part of the DMN. It has been repeatedly noted that the DMN is suppressed during attention demanding tasks (Whitfield-Gabrieli and Ford, 2012) and show anticorrelation with DAN. It has also been proposed that the intrinsic activity of DMN is organized into regions representing readiness for action (Raichle, 2015b). In schizophrenia, the DMN regions often show hyperactivation with reduced suppression during tasks, which has been associated with cognitive impairments (Garrity et al., 2007; Jeong and Kubicki, 2010; Meyer-Lindenberg et al., 2005; Sharma et al., 2018). Psychiatric disorders such as schizophrenia (Sharma et al., 2018; Whitfield-Gabrieli and Ford, 2012), major depressive disorder (Anticevic et al., 2013), and bipolar disorder (Vargas et al., 2013) are characterized by widespread disruptions in brain connectivity, which may impair inter-regional communication and reduce network efficiency. Resting fMRI is also being used in machine learning-based prediction of psychiatric disorders (Lei et al., 2022; Lewis et al., 2025b). Task-based FC analyses have also linked specific brain networks to particular cognitive functions and symptoms of psychopathology. Review of extensive findings is beyond the scope of this paper.

Despite its utility, sFC is limited by its inability to capture the temporal dynamics of cognitive processes. For example, during an N-back task, participants engage several cognitive processes such as attention, instruction registration and recall, stimulus visualization, decision-making, response execution, error monitoring, and error correction. Each of these processes require dynamic reconfiguration of functional network for optimal execution. Averaging BOLD responses across the entire task period obscures these processes. Additional challenges include physiological noise (e.g., respiration, cardiac activity), ambiguities in coupling dynamics (Buckner et al., 2013), and the potential for spurious effects (Siddiqi et al., 2022). These limitations reduce biological and clinical interpretability and translational value of FC findings, particularly for neuromodulation (Isserles et al., 2021).

### 2.3. Dynamic Functional Connectivity

Dynamic functional connectivity (dFC) analyses are increasingly being used to capture the changing patterns of neural coordination over time, providing insights into how brain networks reorganize in response to discrete stimuli (Rolls and Deco, 2011; Wang and Krystal, 2014). The dFC methods address limitations of sFC by recognizing that the functional networks adapt by changing the network architecture while responding to the stimuli (Chang and Glover, 2010). This temporal variability, or non-stationarity, is a fundamental property of neural systems. Empirical evidence supports substantial fluctuations in FC over short timescales during both rest (McIntosh et al., 2008) and task performance (Garrett et al., 2011) conferring computational advantages for neural processing that is leveraged by the dFC compared to static networks (Li et al., 2017). However, the degree of FC variability may differ across brain subsystems and clinical populations. In schizophrenia, reduced variability in the DMN and increased variability in visual and subcortical regions (Zhang et al., 2016) and frontoparietal and temporal areas (Ma et al., 2014) has been observed. These findings suggest that distinct cognitive functions may require varying degrees of FC flexibility over time further justifying the use of dFC in investigating brain functions (Debener et al., 2006; Fox et al., 2005; Garrett et al., 2011).

The sliding window approach is the most widely used dFC method that computes correlation matrices within a fixed-length temporal window that is incrementally shifted across the time series (Handwerker et al., 2012; Hutchison et al., 2013; Sakoglu et al., 2010) (Figure 1, Bottom). This method has revealed clinically relevant patterns, such as prolonged dwell time in sparsely connected states and reduced recurrence of highly interconnected states in depression (Zheng et al., 2022) and increased time in hyperconnected visual states and hypoconnected DMN states with reduced time in globally connected configurations in psychotic-like experiences (Barber et al., 2018). However, sliding window method faces methodological challenges including a lack of consensus on optimal window length and shift intervals, limited sensitivity to abrupt changes in connectivity, statistical uncertainty in fluctuation detection (Hindriks et al., 2016; Lindquist et al., 2014; Zhang et al., 2018), and poor test-retest reliability (Zhang et al., 2018), complicating cross-study comparisons (Hutchison et al., 2013). Although reproducible component selection is made possible by constrained independent component analysis (Lu and Rajapakse, 2005), it does not resolve other limitations (Table 2).

To overcome these shortcomings, alternative methods have been proposed, including dynamic conditional correlation (Lindquist et al., 2014) and dynamic bivariate correlation using weighted graph algorithm(John et al., 2020) with their own distinct advantages and limitations (Choe et al., 2017). One promising alternative is changepoint detection (CPD), which can identify abrupt deviations in time-series data representing statistically significant shifts in the network architecture. This method has been applied in the fields of finance, climatology, and epidemiology (Basseville and Nikiforov, 1993; Krishnaiah and Miao, 1988). The CPD method eliminates the need to predefine window lengths or to shift them at arbitrary intervals (Basseville and Nikiforov, 1993; Li et al., 2019). Recent implementations can detect multiple changepoints in both univariate (Inclan and Tiao, 1994) and multivariate (Fan et al., 2011) data. Still, CPD methods also face limitations. Many rely on binary segmentation, which recursively partitions the time series based on detected changepoints that fails to identify multiple changepoints in complex task-fMRI data and cannot readily localize changepoints to specific brain regions (Anastasiou et al., 2022).

Recent innovations such as the cross-covariance isolate-detect (CCID) method offer promising solutions to the above limitations. CCID applies the Isolate-Detect principle (Anastasiou and Fryzlewicz, 2022) to identify multiple changepoints in the second-order structure of high-dimensional multivariate time series without dimensionality reduction, thereby preserving data integrity (Anastasiou et al., 2022). These advances underscore the growing interest in temporally resolved analyses of BOLD signal dynamics as a means to elucidate the neural basis of cognition and psychopathology. Despite these advances, correlation-based FC remains constrained by its foundational assumption of pairwise independence and its relative lack of mechanistic interpretation. The importance of dFC modeling is that it accounts for non-stationary signal patterns and can identify when important system-wide transitions happen over time. It provokes further investigation into how and why these network-wide changes are occurring and demonstrates the biological relevance of time-resolved modeling. However, dFC cannot provide more refined temporal resolutions at sub-second intervals.

### 2.4. Importance of shorter time scale temporal dynamics of networks

**Figure 2:**
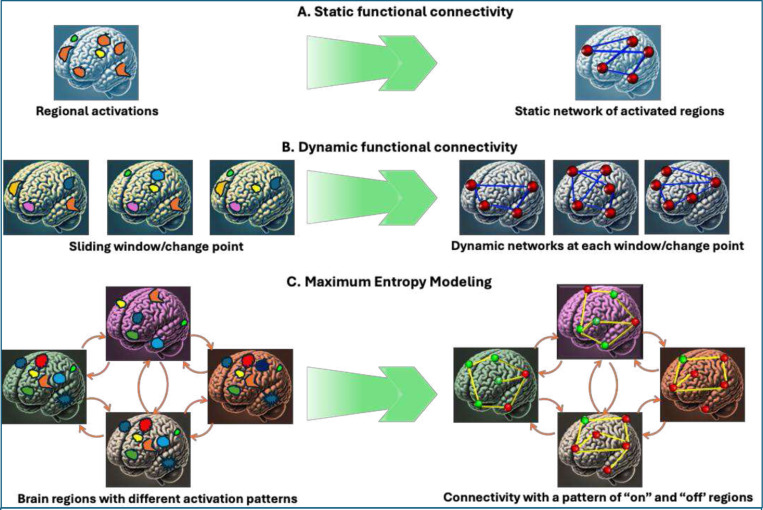
A schematic showing different ways of modeling brain activity. First order model only examines the regional differences in BOLD signals either at rest or while performing a task. A. Static functional connectivity involves using pairwise correlations on the temporally averaged BOLD signals in spatially distinct regions. Temporal averaging is done for the entire scanning session in resting state connectivity, over the task block, or for an event as in task fMRI. B. Dynamic functional network attempts to capture the changes in network architecture over short periods of time. C. The maximum entropy model attempts to identify a pattern of activated or deactivated regions at each TR in a network; the probability of occurrence of these patterns are assigned “energy values.” The energy states derived from these models can be thought of as configurations of neural activity that the brain adopts at each TR.

Brain function is organized across multiple temporal scales, ranging from milliseconds to a few seconds. At the neuronal level, core cognitive processes rely on precisely timed patterns of activity within and across brain regions. Oscillatory synchronization and phase coupling at millisecond scales facilitate effective communication between distributed neural circuits (Fries, 2005; Siegel et al., 2012). Capturing these faster temporal dynamics is critical for advancing our understanding of how neural systems support cognition and behavior. Short-time-scale analyses can reveal transient brain states and network reconfigurations not apparent when signals are averaged over longer periods (Christof et al., 2016). In other words, certain non-significant findings may, in fact, show patterns of changes in brain states within the temporally averaged timeframe, while findings of statistical significance may be enriched by subsumed activation patterns. In addition, traditional approach provides results in 3-dimensions, namely increase or decrease of a hypothesized feature in the anatomical brain space. Shorter time scale dynamics can add the temporal dimension to provide results that are 4-dimenstional in nature. Thus, understanding brain activity in shorter timescales enriches our understanding of neural dynamics in health and disease. Studying temporal dynamics on fine scales supports inference of causal relationships between brain regions through time-resolved measures of effective connectivity (Friston et al., 2003; Lewis et al., 2025a) (Fig 2). Alterations in short-time-scale dynamics have growing relevance for clinical neuroscience as well, since disruptions in oscillatory synchrony are implicated in neuropsychiatric conditions (Uhlhaas and Singer, 2010). These approaches could provide much needed mechanistic insights that are often not captured by sFC or dFC and underscores the translational importance of mapping rapid brain dynamics to better understand pathophysiology and inform intervention strategies.

### 2.5. Comparison with Hidden Markov Models (HMM) and Co-Activation Patterns (CAPs)

While Dynamic FC (sliding window) is the primary comparison often drawn, MEMs also share similarities with Hidden Markov Models (HMM) and Co-Activation Pattern (CAP) analyses. Like MEM, HMMs assume the brain transitions between discrete states. However, HMMs infer states based on transition probabilities without an underlying “energy” topology. MEMs extend this by providing the energy landscape -- a map of stability—which explains why certain transitions are more likely (i.e., the depth of the basin and height of the barrier). Similarly, while CAPs identify instantaneous configurations of high activity, they typically do not model the interaction parameters (J) or the probability distribution of the entire state space as MEMs do.

## 3. Maximum Entropy Models

**Figure 3:**
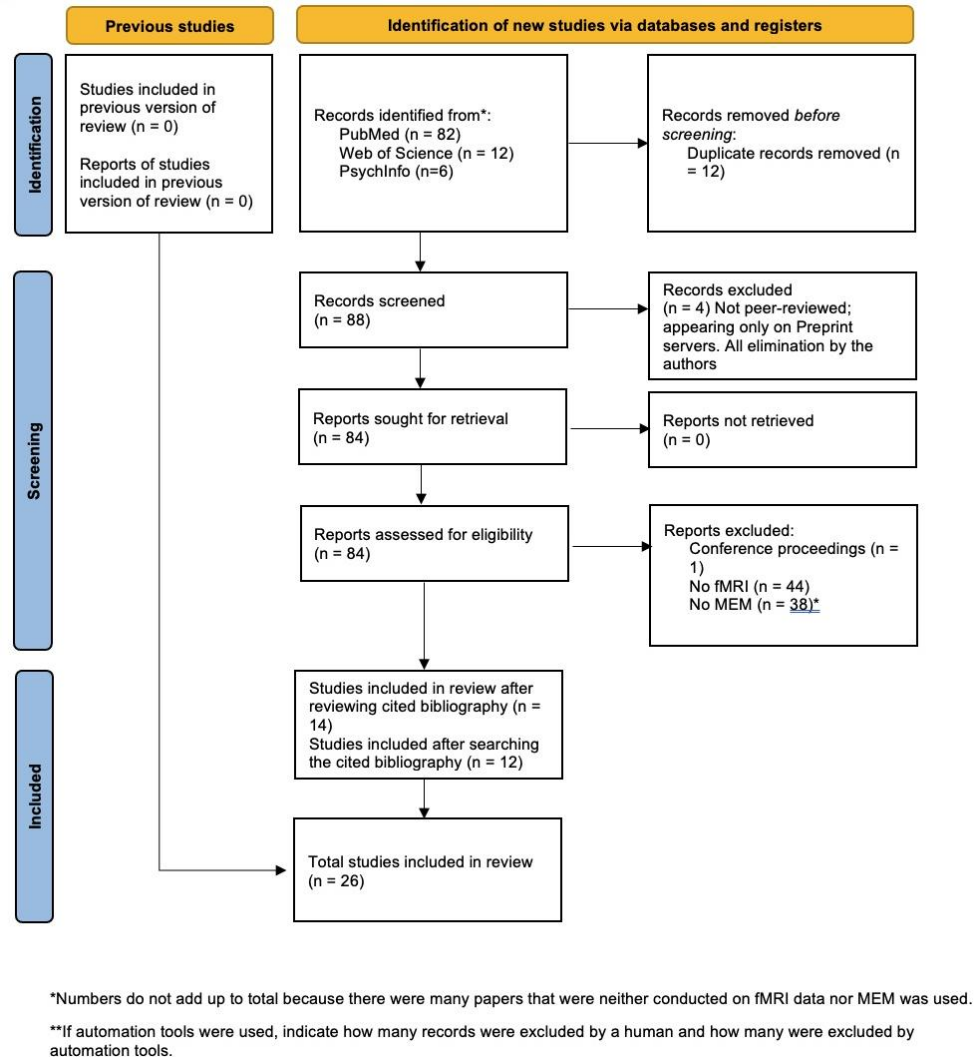
PRISMA flow diagram showing searches of databases and retrieval of papers for review

In order to capture a comprehensive picture of the published literature on the application of MEMs to the fMRI data in neuroscience, we conducted a systematic search of the literature using terms “maximum entropy model”, “fMRI”, “functional magnetic resonance imaging”, :energy landscape analysis”, and “fMRI” in PubMed, PsychInfo, and Web of Science. In addition, we went through the citations in published studies to identify appropriate studies. We excluded conference proceedings, books, and manuscripts appearing on the preprint servers without having been published in a peer-reviewed journal. We also searched MathSciNet using the same terms. Manuscripts that addressed methodological issues without fMRI data were not included in the review. Details are provided in the PRISMA flow diagram (Fig 3). Briefly, using the criteria above, we retrieved 100 publications for review.

After excluding 12 duplicates, we screened 88 and excluded 4 that were on preprint servers only. Out of the remaining 84 publications, we excluded 70 that did not use fMRI data and/or applied maximum entropy modeling and obtained 14 publications for further review. We found additional 12 peer-reviewed publications upon review of cited papers in these publications. Thus, this review includes 26 peer-reviewed publication that met the above criteria. Since these studies addressed different disorders and conditions, and some applied to only healthy persons, the data were not meta-analyzable.

We first discuss basic concepts of MEM including mathematical basis followed by a brief description of practical implementation of MEM to the fMRI data and then main findings from selected papers including neurobiological interpretations of the findings. We also provide a comparative evaluation of MEM-based methods with static and dynamic FC followed by limitations of MEM and future directions in using this method in neuroscience.

### 3.1 The basic concepts of MEM

The Ising model, originally proposed by Ernest Ising in 1925, was developed to calculate the partition function for systems such as magnetic dipoles that can exist as “up” or “down” states. The combined effects of an external magnetic field, random fluctuations, and local interactions among dipoles determine the sum of microstates, which in turn produces macroscale properties like net magnetization and phase transitions in the material (Brush, 1967). While Ising’s original formulation was one-dimensional, it was later extended to two dimensions and then generalized to N-dimensional systems—hence the term “generalized Ising model.” (Ising, 1924; Ising et al., 2017) In neuroscience, the more precise term for this framework is the pairwise MEM. Intuitively, the MEM expresses the probability that a discrete system will occupy any observable state based on its underlying network configuration and external influences. In this analogy, if all dipoles are “spin-up,” there is a net magnetic force, whereas an equal mix of “up” and “down” cancels out macroscale magnetization.

The power of the Ising framework lies in its mathematical generality; the model does not depend on the physical context, allowing dipoles to be replaced with neurons or entire cortical regions. This conceptual flexibility has positioned the MEM as a powerful tool for analyzing fMRI signals and other neural data. The maximum entropy principle ensures that the resulting probability distribution is the least biased one possible while still satisfying empirical constraints. Applied to brain imaging, this approach allows researchers to infer patterns of brain activity directly from observed data, avoiding extraneous assumptions that could misrepresent the underlying functional organization.

MEMs provide a principled framework to simultaneously incorporate first-order (regional activation) and second-order (pairwise coactivation) terms which is one of the major advantages of this method (Figure 3). The first-order terms capture the firing rates of individual neurons or regional activations coding neuronal activity in binary terms (“on” for firing/activation; “of” for silent/deactivation) within a given time interval. In contrast, second-order terms incorporate pairwise interactions between neurons or brain regions representing a more complex population coding scheme. The MEMs quantify network interactions to characterize different patterns of activation or deactivation over time by considering macrostates (behavior of the entire network) as sums of microstates (individual neuronal firing or regional activations and pairwise interactions) and examining how neuronal ensembles or brain regions collectively interact while neurons or regions can be active (“on”) or inactive (“of”) at any moment driven by local interactions. Here, the states are the pattern of on or of conditions of the neurons or brain regions. MEM typically includes up to second-order interactions but can theoretically accommodate higher-order interactions beyond pairwise correlations up to *n*^th^-order models that include interactions among larger tuples of neurons. The framework addresses important mechanistic questions about network behavior under different interaction assumptions (Schneidman et al., 2006). The MEM can be viewed as a generalization of the classic Ising model (Amit et al., 1985) to include first-order as well as second order terms (Yeh et al., 2010). It expresses the likelihood that a system will be in any observable state characterized by pairwise correlations and activation levels at a given time. Unlike conventional FC analyses that do not address collective interaction and are limited by the assumption that pairwise correlations are independent, the MEM explicitly acknowledges non-independence of pairwise correlations (Jaynes, 1957; Schneidman et al., 2003; Yeh et al., 2010). These features of the MEM enable the development of fine-grained mechanistic theories of cognitive impairments, psychopathology, and neuropsychiatric disorders. Since Watanabe and colleagues’ earliest application (Watanabe et al., 2013), many groups have examined structure-function relationships and group differences in psychiatric disorders (Das et al., 2014; Ezaki et al., 2017; Fortel et al., 2022; Jeong et al., 2021; Kang et al., 2021; Theis et al., 2025; Theis et al., 2024).

In terms of building models involving higher-order interactions, several mechanistic and translational questions emerge, such as (a) What does a network derived from pairwise correlations reveal about the behavior of the full set of interactions in the system?, (b) How robust are the inferences drawn from second-order models when compared to those incorporating higher-order interactions, such as triplets or quadruplets?, and (c) If *n*^th^ order models are indeed necessary, how might one address the “curse of dimensionality” that arises when interpreting such high dimensional network data, given the vast number of possible interactions (Schneidman et al., 2006). In the context of the brain, the MEM refers to a statistical model that describes the collective behavior of neurons grounded in the principle of maximum entropy. This principle states that the probability distribution that best represents the current state of knowledge (with the least amount of assumptions) is the one with the highest entropy, subject to known constraints. In other words, it helps to quantify the goodness-of-fit in models of varying degrees of correlations (Schneidman et al., 2003). The widely used pairwise formulation accounts for such interactions in the context of the entire system rather than in isolation. A systematic and hierarchical approach to specifying models of increasing order can elucidate how patterns of network activity across multiple neuronal ensembles depend on the assumptions and constraints of the chosen model inferred in the context of the entire system (Jaynes, 1957). This capacity has made MEMs increasingly attractive in neuroscience, providing a principled framework for investigating the biological relevance of collective neural dynamics and the emergent properties of brain networks.

**Figure 4:**
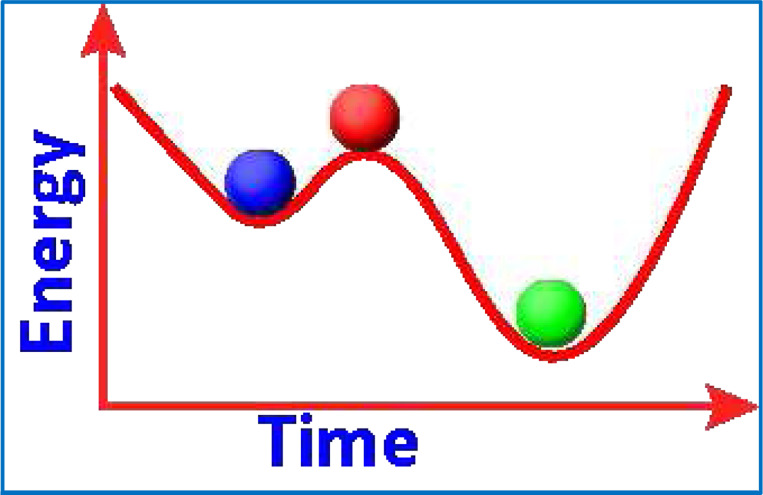
A schematic of a metastable (blue), unstable (red) and multistable (green) states.

Although the MEM is commonly implemented as a statistical approach, it is equally important to consider it from the perspective of dynamical systems theory. From this perspective, the pairwise MEM captures the emergent collective behavior of neural ensembles by characterizing their attractor landscapes, stable, “preferred” network states that may underlie core cognitive functions. The fundamental premise is that specific brain regions become co-activated in recurring patterns over time. Attractor dynamics describes the tendency of neural systems to evolve toward these stable patterns regardless of their initial conditions, generating spatiotemporal dynamics that allow transitions among locally stable states (Deco and Jirsa, 2012). Such transitions can produce multistable states—local minima in the energy landscape (Fig 4), which should not be confused with metastability, where the system can remain in unstable states for prolonged periods.

Unlike purely statistical frameworks, the MEM energy function defines a high-dimensional state space with meaningful dynamical properties: low-energy states correspond to attractors in the system’s phase space, representing stable or metastable configurations (Fig 4). The transition probabilities between these states, shaped by energy barriers, provide insights into the system’s dynamics that conventional FC analyses cannot offer (more in Section 3.2). In addition, MEMs can predict both the probability distribution of neural states and their transition trajectories over time (Kim, 2018; Schneidman et al., 2003). Moreover, the resulting energy landscapes exhibit critical phenomena such as phase transitions and metastability—hallmarks of complex dynamical systems (Greicius et al., 2003). By identifying local minima and the barriers separating them, the MEM provides a mechanistic framework for understanding how the brain dynamically navigates its repertoire of functional states in response to internal demands and external stimuli. This approach aligns with theoretical work proposing that cognition emerges from structured transitions among metastable brain states (Gu et al., 2018; Meshulam et al., 2017), positioning MEM as a bridge between statistical physics and nonlinear dynamics in neuroscience.

Clinical significance of metastability has been demonstrated in different studies. One study proposed metastability as a mechanistic biomarker after examining the HCP-EP and COBRE data on schizophrenia and was able to classify cases from controls with moderate accuracy(Hancock et al., 2023). Alzheimer’s disease patients showed decreased global metastability compared to persons with mild or subjective cognitive impairment (Cordova-Palomera et al., 2017). Similarly, metastable dynamics in conjunction with transient amplitude modulation of the EEG signals was associated with autistic-like traits in healthy individuals(Sase and Kitajo, 2021). In addition, metastable neural dynamics were associated with performance on various cognitive tasks in healthy persons. For example, while higher metastability in cognitive control networks was associated with task-based reasoning, when novel problem solving was needed, fluid intelligence, and when sensorimotor networks were associated with low metastability (Alderson et al., 2020).

The MEM has been used widely in neuroscience to investigate collective activity of neuronal populations, providing a powerful strategy for examining how the brain may optimize information processing by maximizing entropy under relevant constraints (Keshmiri, 2020; Yeh et al., 2010). This implies that neural activity is organized to be maximally unpredictable within structural and functional constraints to support an effective strategy for efficient coding and information transmission. Grounded in information theory, this principle holds that the brain’s immense complexity enables diverse neural activity patterns, supporting flexible and efficient information encoding. According to efficient coding theory, an extraordinarily complex network and greater diversity in firing patterns increases the system’s capacity for information processing while simultaneously minimizing resource expenditure and redundancy (Barlow, 2012; Laughlin, 1981). By maximizing entropy, the brain can distribute information processing across distinct neuronal ensembles, thereby supporting robust and adaptive function.

Nevertheless, the brain’s capacity for information processing is constrained by functional demands, intrinsic computational limits, metabolic costs, structural wiring constraints, and the environmental context (Simoncelli and Olshausen, 2001). Within this framework, MEMs provide a structured approach to estimate neural activity patterns that emerge under these constraints, offering insights into how the brain computes, encode memories, and generate behavioral outputs. MEM formalism accomplishes this by constructing a probability distribution that maximizes entropy while satisfying empirical constraints of the data. Notably, MEMs often show that the statistics of neural states can be approximated using only first- and second-order interactions, as pairwise dependencies frequently suffice to model complex biological networks (Merchan and Nemenman, 2016; Wilsenach et al., 2022). Nonetheless, understanding higher-order interactions remains an important area of research, as these can further illuminate the collective dynamics of neural systems.

### 3.2 Mathematics of the MEM

The entropy H(P) of a discrete probability distribution P over a set of possible brain states $\left(x_{1},x_{2},\;\ldots ,\;x_{n}\right)$ is defined as:

(1) $$H(P)=-\sum_{i=1}^{n}P\left(x_{\text{i}}\right)\log P\left(x_{\text{i}}\right)$$

where $P\left(x_{i}\right)$ is the probability of observing brain states $x_{i}$ and n is the total number of possible brain states. This quantity H(P) measures the uncertainty or randomness in the distribution. A higher entropy indicates greater uncertainty about which state will occur.

The goal of MEM is to find the probability distribution that maximizes this entropy value while satisfying constraints derived from one’s experimental observations. These constraints typically come from measurements of brain activity patterns in fMRI data.

For brain networks, these constraints often include:

First-order constraints: The average activation rates of individual brain regions

(2) $$\sum_{i=1}^{n}P\left(x_{\text{i}}\right)\sigma_{\text{j}}\left(x_{\text{i}}\right)=\langle \sigma_{\text{j}}\rangle$$

Second-order constraints: The correlations between pairs of brain regions

(3) $$\sum_{i=1}^{n}P\left(x_{\text{i}}\right)\sigma_{\text{j}}\left(x_{\text{i}}\right)\sigma_{\text{k}}\left(x_{\text{i}}\right)=\langle \sigma_{\text{j}}\sigma_{\text{k}}\rangle$$

Here, $\sigma_{j}\left(x_{i}\right)$ represents the state (active or inactive) of brain region j in global state $x_{i}$, while $\langle \sigma_{j}\rangle$ and $\langle \sigma_{j}\sigma_{k}\rangle$ represent the observed average activation of region j and the observed correlation between regions j and k, respectively.

To solve this constrained optimization problem, one can use the Lagrange multipliers method. This yields a probability distribution of the form:

(4) $$P\left(x_{\text{i}}\right)=(1/Z)\exp\left(-E\left(x_{\text{i}}\right)\right)$$

where $E\left(x_{i}\right)$ is the energy of state $x_{i}$, given by:

(5) $$E\left(x_{\text{i}}\right)=-\sum_{j=1}^{n}h_{\text{j}}\sigma_{\text{j}}\left(x_{\text{i}}\right)-\sum_{j=1}^{n}\sum_{k>j}^{n}J_{\text{j k}}\sigma_{\text{j}}\left(x_{\text{i}}\right)\sigma_{\text{k}}\left(x_{\text{i}}\right)$$

In fMRI applications, this ‘energy’ represents a statistical likelihood of occurrence activation patterns, not metabolic energy. The parameter $h_{j}$ represents the bias term for each brain region j. The parameter $J_{jk}$ represents the coupling strength between brain region j and k. Z is the partition function, a normalization constant that ensures the probabilities sum to 1:

(6) $$Z=\sum_{i=1}^{n}\exp\left(-E\left(x_{\text{i}}\right)\right)$$

This formulation creates a direct relationship between energy and probability: states with lower energy have a higher probability of occurrence, while states with higher energy occur less frequently. Energy assessed using the MEM is an interpretable unitless scalar quantity because it represents the likelihood of the system being in certain configurations of on/of network states, with lower energy corresponding to occurrence of higher probability network configurations and vice versa. Energy statistically describes the collective behavior of several brain regions at a given point in time. In the context of MEM, this energy term is a mathematical construct, rather than a physical quantity like in thermodynamics. Further, higher energy states do not necessarily mean higher “activation” because nodes in a network can all be in the ‘of’ state that can occur at relatively low probability, thus having high energy compared to other states in the system. In our studies, we have found all of and all on states were more frequent than other states (Theis et al., 2025; Theis et al., 2024).

**Figure 5.**
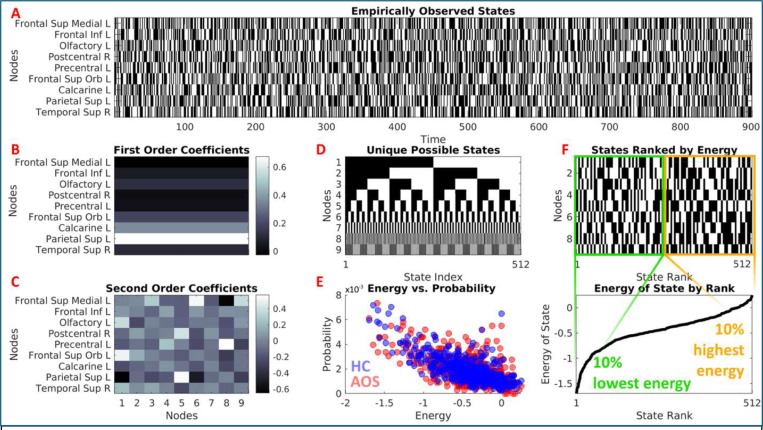
Beginning with a region-by-region (nodal) binary representation of the fMRI activity, called the empirically observed states (A), an MEM is it. The MEM consists of first-order coefficients (h values) (B) and second-order coefficient (J values) (C) which determine the probability of occurrence for each unique possible state (D). Energy and probability are inversely related (E) and can be examined in a group-wise fashion if different MEMs are it per group. States can then be ranked according to their energy (F), and group-wise changes in parameters and energy-state distributions compared, such as between adolescent onset schizophrenia (AOS) patients and demographically matched healthy controls (HC)

The model’s energy value can be interpreted as the stability of brain states, with lower energy states corresponding to more stable configurations that the brain naturally tends to adopt and visit more often. This parallels physical systems described by statistical mechanics, where the Boltzmann distribution governs the probability of different energy states. Thus, the MEM defines the probability distribution that maximizes entropy given a set of constraints. The solution to this problem is an exponential family distribution, which is often used in various fields, including neuroscience, physics, and machine learning, to model complex systems in a principled way. MEMs have been applied in several ways to understand brain function, particularly in the study of neural activity and networks. One example application is given in (Figure 5) from our study on adolescent onset schizophrenia (AOS) (Theis et al., 2025).

### 3.3 Practical Implementation of MEM for fMRI

The analysis of fMRI data typically begins by converting continuous BOLD signals to binary states using an appropriate threshold. Each region is classified as active (+1) or inactive (0) at each time point (TR) using a threshold, which usually uses z-scoring of BOLD signals. The validity of binary thresholding is a critical methodological choice. While binarization simplifies the continuous BOLD signal, it improves the signal-to-noise ratio for detecting state transitions. Common thresholding strategies include using the mean BOLD signal (treating values >0 as “on” and <0 as “of” after z-scoring) or a specific percentile cutoff (e.g., top 15% activity). However, care must be taken: thresholds that are too high may artificially stabilize the “all-of” state, while thresholds that are too low may obscure distinct patterns (Theis et al., 2025; Watanabe et al., 2013). From these binary activations, which contain many repeating entries due to the underlying probability distribution of occurrence of the binarized activation states, activation rates for each brain region and the empirical pairwise correlations between regions are calculated. The matrix of all possible state vectors, each represented as $V_{k}$, where the index k runs from 1 to $B=2^{N}$ where N=number of regions in the network and each $V_{k}$ represents an “on/of” pattern of nodal activity. Energy, E, is defined for each possible state, $V_{k}$, according to the pairwise MEM(Yeh et al., 2010):

(7) $$E\left(V_{k}\right)=-\sum_{i=1}^{N}h_{i}\sigma_{i}\left(V_{k}\right)-\left(\frac{1}{2}\right)\sum_{i=1}^{N}\sum_{j=1.j\neq i}^{N}J_{ij}\sigma_{i}\left(V_{k}\right)\sigma_{j}\left(V_{k}\right)$$

where the N-by-N matrix J and the N-vector h are parameters to be determined from the imaging data using a fitting algorithm. Each term $\sigma_{i}\left(V_{k}\right)$ takes the value 1 if region i exhibits suprathreshold activation in state $V_{k}$ and takes the value 0 otherwise. While the energy value itself has no absolute interpretation and only relative changes in energy are meaningful, the energy of a state maps to the probability of a state, $P\left(V_{k}\right)$, as follows:

(8) $$P\left(V_{k}\right)=exp\left(-E\left(V_{k}\right)\right)/\sum_{i=1}^{B}exp\left(-E\left(V_{i}\right)\right)$$

Through iterative fitting procedure such as gradient descent, parameters $h_{j}$ and $J_{jk}$ are determined to match observed statistics.

Once fitted, the model provides several valuable quantities such as each region’s activity level, h, pairwise functional interactions, J, energy of the states, the energy landscape showing probable brain states, and entropy quantifying overall variability in brain states. These parameters provide a more accurate representation of the complex patterns of brain activity that underlie cognitive processes and neuropsychiatric disorders (Ezaki et al., 2017; Gu et al., 2018; Theis et al., 2025).

#### Software Implementation:

Several open-source toolboxes are available for implementing MEM and ELA. The **Ezaki toolbox** (MATLAB) is widely used for fitting pairwise MEMs and calculating disconnectivity graphs. Python implementations are also increasingly available in repositories associated with recent publications (e.g., the *maxent_toolbox* or custom scripts from the Human Connectome Project analyses).

##### Energy landscape analysis (ELA)

An energy landscape is a visualization technique for MEM. It represents a global statistical structure of network configurations. ELA is a data-driven method for analyzing fMRI data by fitting an Ising model to characterize brain dynamics as movement on an energy landscape. Analysis of the energy landscape provides both quantitative metrics and qualitative perspective of all states and their associated energies, as well as analyzable quantities. The energy landscape represents global statistical structure of probability distributions of network configurations, offering theoretical bridges between observed activity and underlying physical principles. The MEM implementation assigns energy values to all 2^*N*^ possible activity patterns, after which the landscape is constructed. It is possible to examine the trajectory of transitions of these states and relate them to cognitive tasks. There are at least two popular ways to define the energy landscape, a Hamming-distance based method and a principal component analysis (PCA)-centric method.

In the Hamming approach, the first step is to identify all “local energy minima”. These are activity patterns that have a lowest energy value than all their immediate neighbors (patterns that differ by the state of only one ROI). These local minima represent the brain’s stable states and form the “bottom” of the basins in the energy landscape. Then, the next step is to calculate the “energy barriers” between these local minimums by finding the path between two minima that require overcoming the lowest possible maximum energy point (known as a “saddle point”). This height of barrier represents the “difficulty” of transitioning from one stable brain state to another. These relationships are visualized using a “disconnectivity graph,” which is a dendrogram (tree diagram) showing the local minimums and the energy barriers separating them. Finally, every other (non-minimum) activity pattern is assigned to a “basin of attraction” by following the path of steepest energy descent—repeatedly moving to the lowest-energy neighbor—until a local minimum is reached. The resulting multi-dimensional structure, composed of these energy basins, the local minimums they lead to, and the energy barriers between them, is the final energy landscape using the Hamming method (Fig 7, bottom panel).

The PCA approach to define an energy landscape seeks to simplify the high-dimensional space of brain state configurations into a 2-dimensional projection, PC1 and PC2, of the empirical brain state occurrence data, and then give this projection a third dimension equal to the energy of each state described by the 2-dimensional PCA coordinates. This method produces a landscape by smoothing surface data between discrete points, and/or averaging of the energies of states if multiple states (of different energies) map to a similar PC coordinate, depending on the landscape image resolution. More than 2 PCs can be used for analysis, but it is convenient to use 2 PCs for visualization.

Regardless of energy landscape method used, common quantities of interest emerge because low energy states are grouped together forming basins. These basins can be measured for depth (or volume in the case of the PCA method), distance apart, or height of barriers separating them. The landscapes can also form the basis for trajectories, which are the temporal movement throughout the landscape. Trajectories are often analyzed in terms of basin dwell times and basin transition rates and can be used as summary quantities for time-resolved brain state dynamics.

A recently published tutorial review provides more details on implementation of ELA (Masuda et al., 2025).

## 4. Biological interpretation of MEM-derived measures

**Figure 6:**
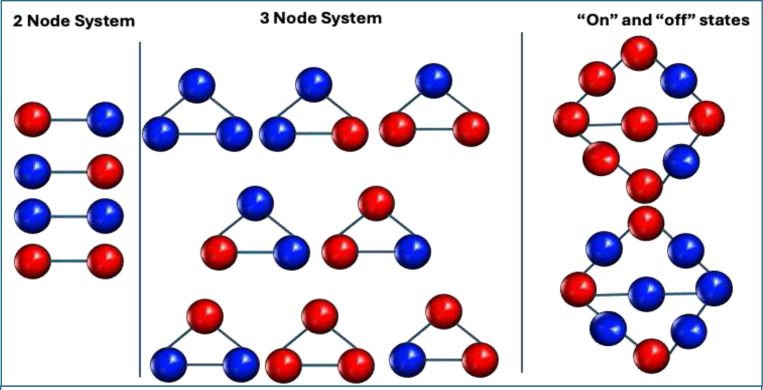
Number of states a given network system can attain. A 2-node system can attain 4 states (left), where node i and j can be in either “on” (Red) or “off” (Blue) states. Similarly, a 3-node system can attain 8 states (Middle panel). The number configurations a network can take increases exponentially ss the number of nodes increase. The panel on the right shows a 9-node network with more nodes in “on” (the network on the top of the right panel) and “off” (the network at the bottom of the right panel) states. When the network is in a high energy state, it is likely to occur with lower probability while a network with low energy state occurs with greater probability.

Although FC is commonly assessed using static or dynamic approaches, these methods do not fully capture the brain’s complexity as an integrative information-processing system. Well-fitted pairwise MEMs can more accurately explain regional interactions because they infer network organization from global activity patterns (Fig 3 and 6). This approach models how activation in one region depends on activity in connected regions, producing network configurations with certain patterns of activated or deactivated regions—called “states”— each with an energy value determined by the occurrence probability of network configurations. Capturing rapid changes in these configurations requires the shortest possible temporal resolution. In the fMRI context, brain states reflect patterns of regions being “on” or “of” within the time window defined by the TR. Some studies have considered each RSN as a node and have modeled multiple RSNs in the same MEM(Kindler et al., 2024; Li et al., 2023). However, this approach can introduce spatial averaging and may affect the interpretability of the results. Although MEMs can also be applied to modalities like EEG, enabling modeling of brain states at much shorter time scales, fMRI provides reasonably good temporal and spatial resolutions while EEG maintains superior temporal resolution.

The principle of maximum entropy has been applied to neuroscience investigations where traditional models have either failed or have been insufficient in providing optimum solution such as neural population coding in retinal ganglion cells (RGCs) and cortical networks, hippocampal place cells, spike train analysis, and FC in large-scale brain networks. A body of literature on neuronal interactions using single neurons and multineuron recordings has shed light on the mechanisms of living neural networks(Yeh et al., 2010). We will first discuss these foundational studies to better elucidate the concepts of MEM.

### 4.1. MEM to examine neural population coding

#### 4.1.1. Spike train analysis for temporal coding

Neurons communicate through electrical signals called action potentials or spikes. The pattern of spikes can be analyzed to understand how the brain processes information. The challenge is the brain’s immense complexity: billions of neurons firing in intricate patterns where one neuron’s activity can excite or inhibit others. Although individual spike trains appear variable, even when the same stimulus is repeated, the overall statistical patterns remain consistent. MEMs allow us to distinguish relatively simple patterns based on firing rates from more complex dependencies that reveal how neurons influence each other. Prior studies show that despite high variability, neuronal responses have highly structured statistical properties suggesting that network dynamics are embedded in these spike patterns (Croner et al., 1993; Shadlen and Newsome, 1998). Using MEMs, it is possible to quantify how much of this structure arises from basic properties like firing rates versus higher-order dependencies to predict the response of a network to stimuli of interest (Cofre et al., 2018; Nasser and Cessac, 2014). The latter papers also describe methods for accounting for spatial distribution of spike trains as well as the impact of memory. Simplified models built on these principles help explain fundamental brain functions such as associative memory, which allows neural networks to link related concepts through their dynamics (Barbieri and Brunel, 2008; Braun and Mattia, 2010) (see later). Specifically, these studies described the attractor dynamics in local cortical circuits supporting memory where one form supports short-term memory while the other one supported long-term memory.

#### 4.1.2. Retinal ganglion cells (RGCs)

One of the earliest and most influential applications of MEMs in neuroscience was the study of RGCs (Simoncelli and Olshausen, 2001). RGCs are neurons in the retina that receive input from photoreceptors and transmit visual information to the brain. Understanding how RGCs encode visual information has long been a central challenge because each RGC integrates inputs from multiple photoreceptors and other retinal neurons to produce a response that represents specific aspects of visual scene such as light intensity, edges, and motion. A key difficulty is that the activity among RGCs is highly correlated, and traditional models often fail to capture this complexity. By applying the maximum entropy principle, Simoncelli et al (Simoncelli and Olshausen, 2001) showed that the collective activity of RGCs could be accurately modeled using only firing rates and pairwise interactions, without overly simplistic assumptions. This approach revealed that the visual system might exploit such pairwise dependencies to efficiently encode information, offering powerful insights into neural coding. Further, MEM was also able to predict the occurrence of specific patterns of neural activity with high accuracy. The model provided insights into how RGCs work together to encode visual stimuli and that the retina may be optimized for efficient coding.

This finding was significant because it suggested that higher-order interactions (e.g., triplet or quadruplet correlations) may not be essential for describing population activity in RGCs. Schneidman and colleagues demonstrated that pairwise correlations alone could reliably predict collective RGC behavior, with most cell pairs showing weak correlations, implying a degree of independence (Schneidman et al., 2006). Several studies showed that RGCs exhibit strong synchronized firings (Arnett, 1978) suggesting that such synchronized firing may play an important role in processing and transmitting visual information(Meister, 1996) based on recordings from pairs of neurons. Further studies on collections of 7–10 neurons showed that pairwise interactions can largely explain synchronized firing. MEM was helpful in investigating larger ensemble of neurons that showed that in primate RGCs, more than 99% of the spatial correlation structure (pairwise interactions between neighboring cells) could be captured by a second-order model and the activity of individual cell was weakly redundant (Shlens et al., 2009). This success indicates that much of the complexity in RGC populations can be explained by relatively simple models, challenging the notion that complex neural interactions always require complex representations. However, some studies suggest that higher-order terms may still be necessary under certain conditions to fully capture population dynamics. Importantly, principles developed through MEMs in RGC studies have provided a general framework for understanding neural coding across other brain systems.

#### 4.1.3. Hippocampal Place Cells

In the hippocampus, place cells are neurons that become active when an animal is in a specific location (O’Keefe and Dostrovsky, 1971). MEMs have been used to analyze the joint activity of place and non-place cells to understand how the spatial information is encoded. By treating these cells as an interacting network, studies have examined how collective behavior supports place-modulated activity, with theoretical models suggesting attractor dynamics as an underlying mechanism (Fuhs and Touretzky, 2006; Kali and Dayan, 2000; Samsonovich and McNaughton, 1997). A recent study applied MEM to model the probability of all possible joint activity patterns in about 80 CA1 neurons of awake mice. Remarkably, mean activities and pairwise correlations were sufficient to accurately predict higher-order network phenomena and individual cell activity (Meshulam et al., 2017). Interestingly, modeling the entire population together yielded better predictions than analyzing place and non-place cells separately. This work showed that the hippocampal spatial map can be described by a set of low-energy (high-probability) states, suggesting that the brain may use an efficient coding strategy for spatial representation. Notably, despite the modest population size (~80 neurons), the MEM predicted the full probability distribution without overfitting, highlighting its potential as a viable framework for modeling population-level neural activity.

### 4.2. Cortical Networks: Primary visual cortex analysis

The MEM has been applied to study activity patterns in the primary visual cortex (V1), extending beyond small neuronal populations. By modeling the joint activity of neurons, it was found that MEMs could predict specific patterns of neural population activity, though performance was somewhat lower than in the retina: 88±7% of neural correlations in V1 cortex compared to 90–99% for the retinal cells (Tang et al., 2008). Another study reported that in a 10-neuron visual cortex system, about 93% of the neural activity was accounted for by pairwise interactions while 7% required higher order interaction (Yu et al., 2008). These studies were conducted on anesthetized animals. Investigation of visual and prefrontal cortical neurons during wakefulness and sleep showed that pairwise interaction in V1 and V4 explained over 90% of activity patterns in both states, but this did not hold for dorsolateral prefrontal cortex (DLFPC), which required higher order correlations regardless of the condition (Chelaru et al., 2021) suggesting that in cellular studies, MEM successfully predict a large fraction of activity patterns in unimodal cortex, while higher order modeling may be needed in cells in the polymodal regions that are downstream. A subsequent study that rigorously addressed error estimates reported that the MEMs explained 80–95% of pairwise activity but excluding inhibitory neurons overestimated the synchrony (Zanoci et al., 2019). Studies of larger cortical populations showed that MEMs performed well for small ensembles (~20 neurons) but became less accurate beyond 25 neurons, necessitating higher-order interactions (Olsen et al., 2024). For example, a system of 25 neurons can have 2^²⁵^ possible states (over 33 million), that may explain why pairwise models may become insufficient at larger scales.

Nonetheless, these findings suggest that in certain regions and ensembles, simple firing rates and pairwise correlations can account for most population activity, implying the brain may simplify complex interactions to facilitate efficient processing. In larger networks, however, higher-order terms often need to be included. Variants of the MEM have improved performance by adding constraints on the probability of a given number of neurons being active (Tkacik et al., 2014) or silent (Shimazaki et al., 2015) and include higher order correlations. Considering the findings on hippocampal place cells and cortical neurons, it suggests that application of MEM to mixed population of cells, as in the tissue, can explain large proportion of activity patterns rather than a population of one type of cells. Overall, the pairwise MEM and its extensions provide a powerful statistical framework for predicting network responses and show that simple interactions often capture much of the temporal structure in neural spike trains, indicating that neural coding may be more straightforward than previously assumed.

In summary, MEMs have been applied to analyze neural population coding in various brain systems. First, MEMs help decode temporal coding in spike trains by distinguishing simple firing rates from complex inter-neuronal dependencies, revealing that structured statistical patterns underlie seemingly variable neuronal responses. In RGCs, MEMs show that pairwise interactions largely explain collective activity and synchronized firing, challenging the need for higher-order models while highlighting the retina’s efficient coding strategy. For hippocampal place cells, MEMs predict spatial representations using low-energy network states and demonstrate that modeling the entire population together yields better predictions than analyzing cells separately. In the primary visual cortex, MEMs account for most activity patterns through pairwise interactions, though larger and more complex networks require higher-order constraints. Overall, MEMs and their extensions provide a robust statistical framework for understanding how simple interactions can capture much of the neural population dynamics in different brain regions, suggesting that neural coding may often be more straightforward than previously thought.

### 4.3. From Cell Populations to Functional connectivity

The precise biological cascade by which neuronal activity generates the large-scale functional connectivity patterns seen with fMRI remains incompletely understood (Shi et al., 2019). Examining the electrical activity of a population of cells using a proxy measure of these local field potentials poses a big technological and analytical challenge necessitating the need for innovative methods to better understand this phenomenon. The BOLD signal, a proxy for neuronal activity, is largely mediated by astrocytes, which modulate local vasculature to boost blood flow and oxygen delivery. This alters the balance between oxy- and deoxyhemoglobin, producing magnetic susceptibility changes detectable by fMRI (Kim, 2018; Logothetis, 2007). Thus, the hemodynamic response consists of cerebral blood flow, cerebral blood volume, and cerebral oxygen consumption rate. The hemodynamic response function describes these temporal dynamics linking neural activity to BOLD fluctuations. Optogenetic studies with simultaneous fMRI in mice have clarified this further: activating excitatory neurons consistently increases the BOLD signal, while activating inhibitory neurons can produce a biphasic response—an initial increase followed by a decrease—typically only with longer stimulation durations (Moon et al., 2021). Since MEM builds network configurations at each TR, which can range from 0.5s to 3s depending on the experimental design and scanner capabilities, such biphasic response is explicitly included in the modeling unlike the regional activation (first-order models) and functional connectivity (second-order models) that smooth out these variations during averaging. These findings highlight the limits of inferring precise neuronal codes from traditional fMRI analyses, given the BOLD signal’s indirect nature and the modality’s relatively coarse temporal resolution. In this context, statistical-physics-based models such as MEMs may offer distinct advantages for analyzing large-scale brain dynamics, because they do not require explicit biological modeling of unknowns.

Despite these limitations of fMRI, the relationship between underlying neuronal events and the BOLD signal remains robust and well-validated. The BOLD signal, which reflects local changes in deoxyhemoglobin concentration due to neurovascular coupling, reliably tracks aggregate neural activity, particularly the balance of excitatory and inhibitory synaptic inputs. Recent optogenetic-fMRI studies have further substantiated this link, demonstrating that targeted activation of neuronal subtypes yields predictable BOLD responses, thus reinforcing the physiological relevance of fMRI signals for mapping brain function (Suarez et al., 2020).

A critical advancement in systems neuroscience has been the recognition of a strong correspondence between structural brain connectivity, as measured by diffusion MRI (dMRI), and FC. Multiple studies have shown that regions with robust structural connections tend to exhibit higher functional connectivity, as evidenced by synchronous BOLD fluctuations (Suarez et al., 2020). This alignment suggests that the MEM framework is well-suited for modeling macroscale functional connectivity, consistent with its proven utility in capturing population-level activity in systems such as RGCs, visual cortex, and hippocampus. However, this relationship is not purely deterministic; functional connectivity also reflects dynamic, context-dependent interactions modulated by neuromodulatory states, plasticity, and ongoing cognitive demands.

The MEM framework has emerged as a powerful tool for modeling macroscale functional connectivity, precisely because it is grounded in statistical mechanics and captures the collective behavior of large neuronal ensembles without overfitting. The MEM enables researchers to infer the least-biased probability distribution of network states that matches observed constraints, such as mean activity (first-order term) and pairwise correlations (second-order term) and characterize the global organization of brain activity patterns while accounting for the high dimensionality and interdependence of regional signals.

Theoretical and methodological advances have further enhanced the MEM’s relevance for macroscale analyses. Recent work has integrated diffusion MRI-derived structural connectivity as priors or constraints within the MEM framework, improving the interpretability and biological plausibility of functional connectivity models. Additionally, extensions of the MEM now accommodate higher-order interactions, temporal dynamics, and non-stationary network states, allowing for more accurate modeling of large-scale brain dynamics observed in fMRI. Notably, MEM-based analyses have revealed that RSNs exhibit attractor dynamics and metastable states, which the traditional correlation-based methods often overlook.

Taken together, these developments underscore the MEM’s unique strengths for bridging the gap between micro- and macroscale neural phenomena. By unifying insights from cellular-level studies (e.g., RGCs, visual cortex, hippocampus) with whole-brain imaging data, the MEM framework provides a principled, data-driven approach for elucidating the statistical structure of brain activity. This positions MEMs as a key methodology for advancing our understanding of how anatomical connectivity supports dynamic functional interactions across the human brain.

#### 4.3.1: Application of MEM and the Ising model to the fMRI data

**Figure 7:**
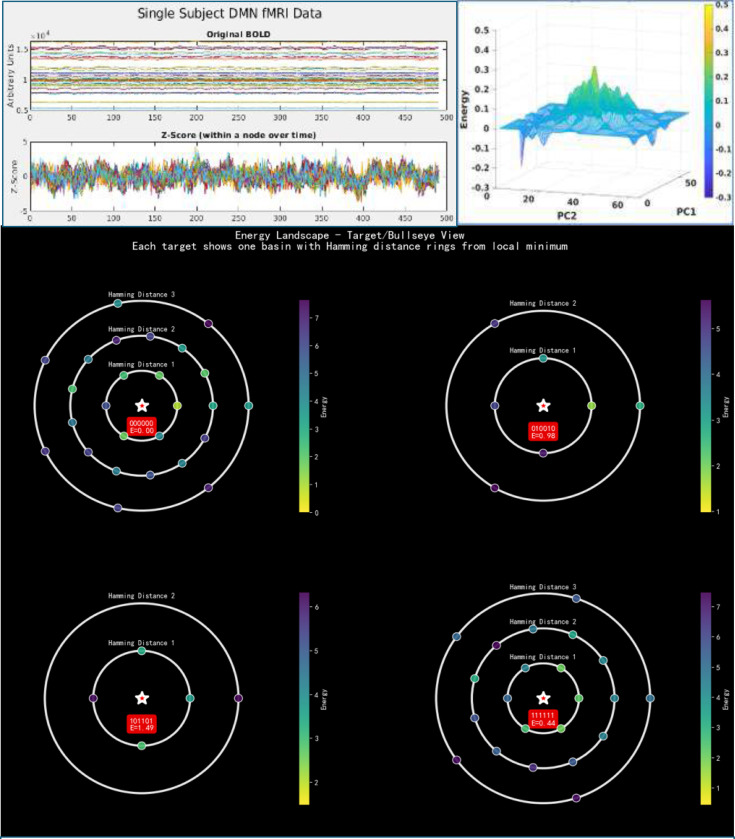
fMRI BOLD signals in 2-dimension (top left) and a 3-dimensional reconstruction of peaks and troughs as a landscape (top right). Bottom panel displays the energy landscape of brain network dynamics, organized by basin of attraction around 4 local minima. Each subplot represents one basin shown as a target/bullseye diagram, where the red star at the center indicates the local minimum state (with its 6-bit binary representation and energy value). Concentric white circles represent Hamming distance neighborhoods from each local minimum, with states positioned on rings corresponding to their Hamming distance (number of differing bits) from the central minimum. The color of each point indicates the energy level of that state, ranging from low energy (yellow/green) to high energy (purple/blue) according to the colormap. This visualization reveals how brain network states are organized in discrete neighborhoods around stable configurations, with energy generally increasing with Hamming distance from local minima. The varying density of states on different rings and the energy gradients within each basin illustrate the complex landscape that governs transitions between different brain network activation patterns.

Although fMRI studies using group differences in regional activations and/or pairwise correlations (Gitelman et al., 2003) have revealed key aspects of brain function at rest and during tasks (Friston et al., 1994a; Friston et al., 1994b; Worsley et al., 1992), some of the fundamental features related to the BOLD data and the analytical techniques call for more innovative methods. In addition to relying on temporal averaging and failing to capture the evolving collective dynamics of neural systems at the systems level, the traditional methods do not consider other features of BOLD signals. Although temporal BOLD signals observed in 2-dimensional plots appear featureless, in a 3-dimensional space, the signals tend to have a pattern that is often missed when the signals are analyzed over longer time scales (Figure 7). The FC studies that use correlations of averaged time-series data misses the detection of these patterns.

MEM, rooted in statistical physics, has been applied to the fMRI time series data in two different ways. Early studies applied MEM to preprocess the fMRI data, but later studies applied it to examine group differences and the influence of underlying white matter connectome on the functional activation patterns. Table 1 gives an overview of studies published using MEM applied to fMRI data. The published studies are grouped as core and foundational applications that deal with methodological advances in applying MEM to fMRI data, followed by studies with clinical implications, and then by a study that examined structure-function integration.

##### 4.3.1a. MEM for fMRI data preprocessing

One of the earliest studies explored applying the Ising model to voxel-wise fMRI as a spatial prior for estimating regional activations (Kim et al., 2000). Kim et al(Kim et al., 2000) noted that the 2^*n*^ state space estimation problem, seemingly NP-complete, can be solved exactly in polynomial time. Here, NP refers to the Neyman-Pearson lemma for comparing competing hypotheses using the likelihoods of the data fitting one hypothesis over the other, which is an alternative to Fisher’s approach of rejecting one hypothesis to accept the other. They argued that the Ising framework could outperform the GLM used in Statistical Parametric Mapping (Friston, 2003) as MEM’s nonparametric approaches require fewer and milder assumptions. They included local spatial priors under the Ising model so as not to assume independence of BOLD signals in the neighboring voxels. The authors tested this on three data sets comparing with GLM on motor, auditory, and visual cortices using different activation protocols for each cortex. GLM showed a number of voxels with extremely low p values, while mutual information-based non-parametric method showed additional voxels with a mixture of spurious and non-spurious activations. However, nonparametric MI with Ising prior removed many isolated activations. The paper’s theoretical premise is interesting and is an important early demonstration of how the Ising model could serve as a spatial prior for voxel-wise activation estimates in fMRI analyses.

While not widely adopted, the Ising model framework continued to be presented for tasks such as spatial partitioning and regularization (Risser et al., 2009). This application pertains to improving the SNR without losing the spatial resolution that could happen with spatial filtering, which is normally used in preprocessing. The authors proposed a more advanced method, Markov Random Field (MRF) that works on unsmoothed data that makes better assumptions about how the activation in neighboring voxels are related to each other. The MRF has a setting, namely temperature that controls how much to assume of the neighboring voxel’s activation is. The paper deals with how to find the perfect temperature setting for all the parcels (the paper used 500 parcels) in an unsupervised setting. These early applications emphasized its utility for improving hemodynamic filter estimates, signal estimation, and managing the large datasets that are typical in fMRI. The method was applied to a sample of event-related fMRI data and demonstrated that unsupervised method was more sensitive to detecting the activations.

Another early approach applied MEMs to rsfMRI to examine whether the resting state of the brain can be compared to any of the known dynamical states by comparing the resting networks from fMRI data with networks derived from numerical simulations. Prior studies had shown certain common features of brain networks such as assortativity of networks, coexistence of large correlated and anticorrelated domains, and large-scale patterns of correlations existing during both rest and activity. Authors proposed that one possible explanation using the concept of critical temperature (Fraiman et al., 2009) meaning that the brain stays near the critical point of second-order phase transition. Critical temperature is the point at which materials undergo phase transitions in physics such as in the evaporation of a liquid to a gas. Conceptually, lowering the temperature deepens the basins and raises the barriers resulting in greater stability with fewer transitions with higher temperature having opposite effects. However, it should be noted that this is not physical temperature but is quantified as relative strength of interactions vs. stochasticity in the binarized representation. Fraiman et al (Fraiman et al., 2009) proposed that the brain operates near an organizational critical point, enabling semi-stable, large-scale activity patterns. This dynamical regime supports a diversity of metastable states (Fig 4) that can flexibly transition to different network configurations as needed, allowing investigators to examine the fMRI time series data from a dynamical system perspective. Such a perspective has major implications for interpreting fMRI time series. Applying MEMs in this way allows researchers to analyze brain dynamics as evolving through metastable states, characterize the regions involved, identify the perturbations required for transitions, and link these network dynamics to behavioral outputs.

Thus, early studies applying the Ising model and maximum entropy models (MEMs) to fMRI data demonstrated their utility as spatial priors for estimating regional brain activations. The Ising model, by incorporating local spatial dependencies among neighboring voxels, was shown to outperform traditional statistical approaches like the general linear model (GLM) in some cases, particularly by reducing spurious activations and making fewer assumptions about the data. Extensions of this framework, such as Markov Random Fields (MRF), allowed for improved signal-to-noise ratios without losing spatial resolution, using unsmoothed data and unsupervised parameter selection. Additionally, MEMs were used to analyze resting-state fMRI, suggesting that the brain operates near a critical point of a phase transition, resulting in flexible and semi-stable large-scale activity patterns. This dynamical perspective on fMRI data emphasizes the brain’s ability to transition among metastable states, providing insights into both network organization and potential links to behavior.

##### 4.3.1b. MEM for fMRI data analysis

###### Core Methods and Foundational Analysis

Prior studies have reported that the patterns of organization of functional networks reflect specific spatial and temporal features to support optimum information processing. Studies using EEG and MEG have reported that such organization exhibited features of “small world network” meaning any two brain regions are connected through only a minimal number of intermediate steps, facilitating efficient communication and integration across the network (Stam, 2004). Such an organization was also observed with fMRI data (Eguiluz et al., 2005; van den Heuvel et al., 2008) further supporting the universality of this organizational principle within functional brain networks. Salvador et al extended the observation to examine the frequency bands of fMRI that showed that the lower frequency time series showed stronger pairwise correlations than higher frequency data (Salvador et al., 2005). This suggests that the temporal dynamics of functional connectivity vary depending on the frequency range analyzed, with lower frequencies potentially playing a more prominent role in coordinating brain activity. Further, FC can show the edges between regions that are not directly connected by white matter fibers, and such regions may show a strong correlation (Adachi et al., 2012). Thus, findings on FC studies need to be carefully interpreted owing to the contribution of above factors, especially because FC poorly reflects the ground truth of connectivity reflected in the structural connections. Research over the last few decades shows that spontaneous activity during rest is not random but instead forms robust spatial patterns known as RSNs (Biswal et al., 1995; Raichle, 2015a), such as the DMN and FPN (Greicius et al., 2003; Raichle, 2015a) which support memory (Raichle, 2015a; Sestieri et al., 2011) and attention (Beckmann et al., 2005; Cole et al., 2010). Watanabe et al demonstrated that the pairwise MEM accurately captures RSN activity and that its functional maps align more closely with anatomical connectivity than traditional FC maps (Watanabe et al., 2013). By inferring local network interactions and primarily on short-range connectivity, pairwise MEMs have shown better correspondence with underlying structural connectomes mapped by dMRI as the ground truth of brain connectivity (Watanabe et al., 2013).

The same group extended the analysis to examine the attractor dynamics of the RSNs. They showed that RSNs exhibit attractor dynamics although this was not directly examined in this study: for example, the DMN had 21 local energy minima—more complex than random graphs—while the FPN was simpler with 4 local minima including a single dominant local minimum, supporting the idea of multistable network states (Watanabe et al., 2014a) supporting prior studies that suggested that resting state networks can be explained by attractor dynamics. Consistent with that Watanabe et al showed that the DMN can have posterior-centric and frontal centric network states whereas FPN was monostable suggesting that the RSNs exhibit different dynamical properties. These studies highlight how the MEM bridges activation rates and co-activity in ways that traditional static and dynamic FC cannot (Honey et al., 2009; Sporns, 2011). These findings mark a turning point, from using the Ising analogy as a preprocessing tool to direct use in fMRI data analyses. This transition underscores the growing recognition of MEMs as a powerful methodology for elucidating the statistical relationships and organizational principles governing functional networks in the human brain.

Understanding such attractor landscapes has important implications for cognitive functions like short-term memory, where a population of local excitatory cortical circuits controlled by inhibitory GABAergic interneurons in the DLPFC maintain stable patterns through positive feedback and long-term potentiation (LTP) (Goldman-Rakic, 1995). The LTP can mediate the activation of the whole set of neurons within the local network when a subset of neurons is activated to retrieve the full memory. Analogously, the state of the network is “attracted” toward the state when the memory formed with “attractor states” within a network (Goldman-Rakic, 1995; Hopfield, 1982) even when a subpart of the memory is presented. Damage to or pathological changes in the prefrontal cortex can impair short-term memory as in schizophrenia (Forbes et al., 2009; Glahn et al., 2005).

Applying MEM to investigate beyond resting networks, the Ising model has been used to map energy landscapes during complex perception tasks requiring attention and memory. Watanabe et al (Watanabe et al., 2014c) showed that bistable visual stimuli produce dynamic transitions between energy minima in the visual area dominant, frontal area dominant, and the intermediate states that also showed distinct associations with the type of perceptions linked to individual brain structure differences. Bistable images refer to the perception of images that can be interpreted in two different ways like in two connected squares that appear like a cube. The visual area dominant minima were associated with the stable perceptions and the frontal area dominant states with activity that correlated with gray matter volume differences of the corresponding brain areas. The importance of this finding is the identification of specific network configurations consisting of a network of regions with higher (on) and lower (of) activations rather than regional activation levels or networks alone.

Pairwise MEM has been applied to understand how the brain organizes its metastable states at rest. To accomplish this, Kang et al., 2017 (Kang et al., 2017) systematically perturbed resting network parameters in three ways: (1) global scaling of interactions, (2) single-node baseline sensitivity changes, and (3) single-edge interaction changes. Effects were measured using total number of local minima and occupation ratios. The baseline MEM identified 18 local minima with high model accuracy (92.6%). The resting state showed maximal number of total local minima compared to all perturbed configurations with relatively equal sharing of occupation time among attractors. Perturbation effects correlated positively with node strength and paired node strength, suggesting that hub regions and hub-connecting edges had greater impact. When both interhemispheric homologous regions were perturbed together, the effects were minimal. However, when asymmetric region pairs were perturbed, the response was dominated by whichever node was more primary. However, coarse spatial resolution of 15 regions ignores subnuclear structure and cortical systems that could be addressed in the future by examining data from ultrahigh-field scanner. In addition, global signal regression remains controversial. The landscape characterization was limited to only total local minima and occupation time metrics with no validation against independent data, task performance, or clinical populations and lack of formal statistical testing in some analyses. Despite these limitations, this study provided important data on subcortical networks exhibiting maximal multistability while remaining poised for reconfiguration, with perturbation effects depending on network topology. This approach offers promise for investigating brain network principles and potentially understanding neurological disorders, though future work should address spatial scale, validate predictions empirically, and connect findings to cognition and behavior.

Ezaki et al (Ezaki et al., 2017) extended the pairwise MEM and energy landscape analyses using pseudolikelihood maximization, minimum probability flow, accuracy indices, and a variation of Dijkstra method to calculate the disconnectivity graph. They implemented these methods on resting fMRI data from two healthy individuals and examined DMN, FPN, and CON. They found that the parameters derived from the likelihood maximization and pseudolikelihood maximization were close to each other. Minimum probability flow could not be applied to CON. The accuracy indices showed that the minimum probability flow deviated from the two maximization methods for the DMN and FPN. They pointed out that this method would be demanding in terms of the amount of data and typical fMRI acquisitions may not be long enough for this method to be applied. This would be even more problematic if larger neural systems with more regions-of-interest be included. However, this method could be better applicable to neuronal spike train data with a high time resolution where length is not a limiting factor as long as the network size is appropriate.

While network size is a limiting factor for MEM fitting, increasing the number of observations, either by increasing the number of scans or the duration of acquisitions is a popular strategy to mitigate this problem. Concatenating data together is also widespread. One study to investigate attractors of landscapes of fMRI attempts to do just this and then relate energy landscape features to network modularity/community structure (Ashourvan et al., 2017). The goal of the study was to understand how the brain facilitates transitions between network states. A group-ICA was run on 20 participants, ages 19–53, with no history of neurological or psychiatric illness, and resting-state components were identified. Regions were assumed to be “proxies” for entire cognitive systems. Energy landscapes were created from a single 10-node MEM fit using concatenated data, in this case 1,190 (TRs) × 20 (subjects) × 4 (runs) = 95,200 samples. Local minima were identified, and basin transitions were calculated. Modular community structure of functional networks was separately determined to provide a classification of basins. The authors conclude that the local minimum of the landscape (the basins) “highlight” three classes of ROIs when compared to the community modularity structure in the resting state. Class-I is described as subcortical, visual, attention, and sensorimotor; Class-II is described as the “putative” executive control network; Class-III is described as the “putative” default mode network and salience network. This paper provides further evidence that the MEM and ELA are technically appropriate for fMRI.

Kang et al (2019) investigated the organizational principles of brain state transitions during rest by applying graph-theoretical analysis to energy landscapes derived from resting-state fMRI data. They used states as nodes, transitions between states as edges, and the transition rate as edge weight. The authors used the pairwise MEM on data from 470 participants in the Human Connectome Project, focusing on 19 cortical regions in the left hemisphere that were binarized into active/inactive states. They identified 14 local minima and constructed state transition networks, a state transition network with all possible states along transition pathways between all pairs of stable states (STN-FS), transition path from all local minima toward global minimum (STN-GM), and transition network comprising of rate-determining transition states and local minima (STN-LM) to analyze transition pathways, rates, and organizational properties. The results revealed that brain states clustered into two main groups, with most inter-group transitions mediated by a hub transition state (TS2) corresponding to the DMN, while the global minimum (local minima 11) represented co-activation of the DMN and prefrontal cortex. When the hub state was removed, transitions occurred via alternate paths suggesting redundancy in transition paths. The analysis demonstrated multi-step transitions with intermediate states, redundant pathways, and more complex, organized transition architecture compared to artificially altered systems. This study offers novel insights into the temporal dynamics of brain state transitions using an innovative network approach. Due to computational constraints, the analysis was restricted to 19 cortical regions in one hemisphere, and the neurophysiological interpretation of “active” versus “inactive” states in fMRI remains unclear, particularly after global signal regression.

In summary, recent research demonstrates that the organizational patterns of functional brain networks support efficient information processing, often displaying “small world” characteristics, where regions are interconnected through minimal steps for optimal communication, across modalities like EEG, MEG, and fMRI. FC studies show that these patterns are influenced by frequency, with lower-frequency fMRI signals exhibiting stronger correlations. However, FC can reveal connections between regions not structurally linked, indicating that interpreting FC findings requires caution as they may not always reflect actual anatomical connectivity. The RSNs, such as the DMN and FPN, form robust and functionally meaningful spatial patterns supporting memory and attention. Pairwise MEMs offer a more accurate mapping of these networks and their relationship to anatomical connectivity than traditional FC approaches by capturing the attractor dynamics of RSNs, revealing multistable states (e.g., DMN with many local minima versus simpler FPN), and distinguishing between different dynamic properties of these networks. This approach bridges the gap between activation rates and co-activity, providing insights that static or dynamic FC cannot. The concept of attractor landscapes has important implications for cognitive functions like short-term memory, where stable neural states support information retention and recall. Applying MEMs to task-based fMRI, such as during perception of ambiguous images, has linked specific network configurations to types of perception and individual brain structure. The MEMs have also been applied to study the brain’s metastable states at rest, showing that perturbations to network parameters affect the number and occupation of local minima, with hub regions playing a key role in network stability and flexibility. While technical challenges remain, such as limitations in network size due to data requirements and the need for longer scans or data concatenation, studies have confirmed the appropriateness of MEMs and ELA for investigating brain network organization. This methodology has revealed that local minima in the energy landscape correspond to distinct functional communities and that brain state transitions are mediated by hub states with redundant pathways, providing a nuanced view of how the brain dynamically reconfigures itself. Overall, these findings highlight the power of MEMs to move beyond simple preprocessing tools, offering a framework to elucidate the statistical, structural, and dynamic principles governing brain networks in both health and disease.

###### MEM Estimation Advances

Kang et al (2021) developed a Bayesian estimation method (BMEM) for the pairwise MEM to enable individual-level ELA of brain states from resting-state fMRI data with limited sample sizes. Using simulated data with 8- and 15-ROI networks and empirical HCP data (468 participants, 15 subcortical/limbic ROIs), the authors evaluated BMEM performance against conventional maximum likelihood estimation (MLE) across varying sample sizes (50–1,200 timepoints) and prior types (zero-mean, group-average, group-concatenated). BMEM with empirical priors from group-concatenated data showed substantially lower root mean square error than MLE or zero-mean priors for small samples, with accuracy comparable to MLE for larger datasets. The estimated MEM parameters and energy landscape features demonstrated high subject specificity (inter-individual similarity correlation with functional connectivity: r=0.88 for MEM, r=0.86 for energies, r=0.57 for local minima energies), and sparse canonical correlation analysis revealed significant associations between MEM parameters and cognitive scores (r=0.66) and between energy landscape features and cognition (r=0.46), comparable to functional connectivity (r=0.64). While BMEM successfully addresses the data-hungry nature of traditional MEM estimation and enables clinically feasible individual-level analysis, important limitations include heavy dependence on prior quality (particularly problematic for very short time series), and use of an arbitrary prior precision parameter rather than data-driven hyperparameter selection. The study focused on analysis of a relatively small number of subcortical networks and did not validate on clinical populations despite potential applications to characterizing brain disorders mentioned throughout the paper.

Jeong et al (2021) proposed the another analytical approach, namely Variational Expectation-Maximization-based MEM (VEM-MEM), an empirical Bayes estimation approach designed to overcome the critical limitation of the pairwise MEM, namely, its unreliable parameter estimation when using the small sample sizes typical of individual rsfMRI scans. VEM-MEM’s core method is a hierarchical Bayesian framework that automatically and empirically incorporates group information as a robust prior within a variational EM algorithm to estimate individual pairwise MEM parameters. Simulation results demonstrated that VEM-MEM significantly outperformed conventional MLE, especially with short time series and larger network sizes, yielding dramatically lower estimation errors. In an experimental study comparing children with ADHD and typically developing children (TDC), the method showed significant group differences in nonlinear dynamic properties, such as the occupation times of stable brain states (local minima) within the DMN and Executive Control/Salience Network (ECN+SAN). Furthermore, pairwise MEM features were found to be more sensitive to group differences and better correlated with ADHD behavioral scores than traditional Pearson correlation-based FC. However, the authors acknowledge that the application is currently limited to smaller subnetworks (around 10–20 nodes), and the challenge of reliably extending this individualized ELA to larger-scale brain networks due to exponentially growing state dimensions still needs further research.

Khanra et al (2024) examined the test-retest reliability of ELA. Using fMRI data from the Midnight Scan Club (MSC; 8 participants, 10 sessions each) and HCP (87 participants, 4 sessions each), the authors constructed a permutation test to assess whether energy landscape indices were more consistent within the same participant across sessions than between different participants. They quantified energy landscape similarity using four discrepancy measures: interaction strength, activity patterns at local minima, activity patterns averaged over attractive basins, and normalized branch length (dL). The results showed significantly higher within-participant than between-participant reliability across all four indices for whole-brain networks (N=7 ROIs), default mode network (DMN; N=8 ROIs), and cingulo-opercular network (CON; N=7 ROIs), with p<0.001 for most comparisons using both conventional likelihood maximization (requiring concatenation of 4 sessions) and variational Bayesian approximation methods (enabling single-session analysis). While this work provides valuable evidence for individual-level energy landscape fingerprinting, several limitations warrant consideration: the permutation test’s statistical power depends heavily on arbitrary choices of sample sizes for averaging (80–100 samples), the study excluded two MSC participants due to data quality concerns which may bias reliability estimates upward, the variational Bayesian method showed somewhat weaker reliability than conventional methods despite higher accuracy of ft, and the discrepancy measures are multidimensional and difficult to relate to standard reliability metrics like ICC.

Using 1,002 healthy subjects from the S1200 HCP release, Hosaka et al (2025) sought to examine how fMRI signal properties relate to MEM/ELA features like basin transitions. The authors also created different “surrogate” datasets that are based on real data but randomized or altered to remove specific statistical relationships in the real data. While landscapes were not shown, the authors find that landscape shape and basin transition patterns are largely explained by “stationary and linear statistical properties” of the fMRI signal, and that MEM/ELA “cannot extract nonlinear and complex dynamics.” The article thus offers tepid criticism of the method. It suggests the prospect of calcium imaging to determine “which simple models describe macroscopic resting-brain dynamics” best. However, as already discussed in this review, the MEM has long been used in cellular neurophysiology experiments.

Another study examined an important question regarding the structural connectivity and energy constraints on the patterns of connectivity and changes in brain states (Gu et al., 2018). MEMs constrained by structural connectivity revealed how network states emerge from anatomical scaffolds (Gu et al., 2018), finding that brain activity samples multiple low-energy configurations, unlike GLM analyses that emphasize only a few regions. In each local minimum, approximately 62% of the regions were active suggesting that the brain activates a large swath of regions forming networks that could provide a diverse set of flexible connections. The distance between the first local minima and the last local minima was an order of magnitude greater than between the first and the second suggesting local minima span a large geographic region in the brain, with most pairs comprised of dissimilar anatomical regions. The activation rates were the lowest in sensorimotor systems while cognitive systems had higher rates and DMN the highest along with strong dissociation in within-system dynamics from the between-systems dynamics. A related question was examined by Ashourvan et al (2021) who used dMRI and intracortical EEG (iEEG), instead of fMRI, to elucidate that estimating the parameters of the MEM has implications beyond MRI-based functional connectivity. These authors used MEM to understand the quantitative relationship between structural connectivity measured using dMRI and emergent brain activity using iEEG. Here, five patients with refractory epilepsy were studied and MEMs were fit to hundreds-of-thousands of samples (recordings were at 512 Hz). An ROC analysis was used to measure the similarity of MEM-predicted connectivity (through the J parameter) and the MRI-derived structural connectivity. The area under the curve for pairwise MEM J *was consistently* as high as around 0.9, and log streamline count versus J correlations were between r=0.4 and r=0.6. These values were comparable, but arguably slightly better at predicting structural connectivity as compared to using iEEG partial correlation, demonstrating that the MEM can predict structural connectivity as well or better than second-order-only methods. The use of iEEG and dMRI makes this study particularly useful. It demonstrates that the MEM framework is appropriate for multi-modal techniques and can provide a basis to related electrophysiology to structural connectivity.

To summarize, the recent advances in MEM estimation for functional brain network analysis address the challenge of limited fMRI sample sizes. Kang et al (2021) introduced a BMEM approach that leverages group-level priors to improve parameter and energy landscape accuracy at the individual level, showing high subject specificity and strong links between MEM features and cognition. However, BMEM depends on prior quality and has not been fully tested in clinical populations. Jeong et al (2021) developed VEM-MEM that automatically incorporates group information, outperforming traditional methods, particularly with short time series and larger networks; it reveals group differences in brain dynamics and better correlates with behavioral measures than standard FC, though it still faces scalability challenges. Khanra et al (2024) demonstrated that energy landscape features are reliable within individuals across sessions, supporting the concept of individual “energy landscape fingerprints,” but noted methodological limitations such as reliance on sample size choices and multidimensional measures. Hosaka et al (2025). found that MEM/ELA mostly reflect stationary and linear fMRI properties, limiting the model’s ability to capture complex brain dynamics. Other studies showed that MEMs constrained by structural connectivity can map how network states emerge from anatomical structure, with significant portions of the brain active in each state and notable differences between systems. MEMs have also been validated with multimodal data (iEEG and dMRI), predicting structural connectivity as well as or better than traditional correlation methods. Overall, these innovations enhance the feasibility, reliability, and interpretability of MEM-based brain network analysis, while highlighting ongoing challenges with priors, network size, and capturing nonlinear dynamics.

###### Clinical Applications

####### Sleep:

MEMs have revealed how RSNs behave differently during sleep stages. Such studies are important because sleep is not a passive process, and the neurobiology of sleep is still not fully understood. Many prior studies using traditional fMRI approaches have had various methodological limitations such as acquiring data at times that would cause circadian misalignment and not acquiring whole night fMRI making it harder for generalization. In a simultaneous resting state fMRI and EEG study that acquired fMRI scans for 55 minutes and then repeated until the subjects were awake or felt discomfort, the application of MEM yielded interesting results. The DMN showed increase in both basal brain activity and connection strength during the slow wave sleep (SWS) but both these measures were decreased during the rapid eye movement (REM) sleep. However, FPN showed opposite pattern during the same sleep stages. Thus, network activity measured by these two parameters were negatively correlated with each other during SWS and REM stages, a pattern not captured by standard FC analyses (Watanabe et al., 2014b).

####### Autism spectrum disorders:

Watanabe and Rees (2017) used ELA to examine how brain activity changes over time in high-functioning adult males with autism spectrum disorder (ASD) using rsfMRI. Autism spectrum disorder (ASD) is a neurodevelopmental disorder characterized by impaired social communication and restricted, repetitive behaviors/interests. Although traditional approaches mapped time-averaged brain activity or connectivity underlying autism-specific symptoms or cognitive abilities, intrinsic fluctuation of brain activity over time have not been fully examined. Authors identified three discrete brain states in both groups. Individuals with ASD showed significantly fewer state transitions than controls. The ELA identified two major states (State 1 and State 2) that were relatively stable (high probability of remaining in the same state) and an intermediate state (State 3) that was relatively unstable (low probability of remaining in the same state). The brain activity frequently transited between State 1 and State 2 via State 3. In the ASD group, all three states were relatively stable, while the intermediate state (State 3) was significantly more stable than among controls due to fewer transitions between State 1 and State 2. The energy barrier for transitions from State 3 was significantly higher in the ASD group than in the control group. These results suggest that the brains of individuals with ASD are characterized by overly stable brain dynamics, which reduces the frequency of state transitions. Further, the indirect transition frequency was negatively correlated with the severity of autism symptoms. Controls showed positive correlation of full-scale IQ with indirect transition frequency, but not in ASD suggesting that ASD is characterized by a different brain-behavior association compared with the control group. Specifically, whereas cognitive ability (IQ) in the neurotypical brain is associated with flexible brain dynamics (that is, frequent state transitions), cognitive ability in the ASD brain is associated with stable brain dynamics. In addition, functional network segregation was significantly increased among ASD compared to controls that was negatively correlated with the frequency of state transitions and positively with the stability of brain dynamics across all participants. These results suggest that the increased functional segregation in ASD contributes to the reduced frequency of state transitions and the increased stability of brain dynamics. These findings provide new insights into the neural basis of autism. The reduced flexibility of brain dynamics in ASD might underpin the difficulties individuals with ASD experience in adapting to changing environments and social situations. Authors suggest that the ASD brain may have developed a different strategy to support cognitive abilities, relying on stable and segregated functional networks. Some limitations of the study include the use of rsfMRI and examining high-functioning adult males with ASD were examined limiting the generalizability of the findings to children and adolescents with more severe autism.

####### Age related changes:

Ezaki et al (2018) analyzed the brain dynamics of the DMN and cingulo-opercular network (CON) in 28 younger (19–30 years) and 28 older (60–85 years) adults using an energy landscape model. They found that in both age groups, the dynamics of both networks were dominated by two main states: s+ (all regions active) and s- (all regions inactive). Older adults visited these two main (s+ and s-) states less frequently. Younger adults showed frequent transitions *directly between* the s+ and s-states. Older adults, in contrast, had more “peripheral transitions”, fluctuations between other, intermediate brain patterns that *did not* involve returning to s+ or s-. Authors created an “efficiency score” (the ratio of main transitions to peripheral ones) that correlated with executive function in the CON but not in the DMN. This score was significantly lower in the older group, suggesting their brain dynamics are less efficient. This implied that with age, brain activity tends to ill-formed fluctuations between intermediate patterns rather than transitioning effectively between the main states of synchronized activity and inactivity. Further, this loss of efficiency was less pronounced in the DMN compared to the CON.

####### Post-stroke aphasia:

A particularly striking clinical finding from the MEM/ELA can be found in poststroke aphasia (Fan et al., 2022). The study follows 16 patients over the course of more than half a year using three timepoints. Within one week after stroke events, patients had language assessment scores that were on average 0.5 out of 1. The MEM/ELA revealed hierarchical brain network re-organization at the end of the 6-month period that corresponded to dramatically improved language assessment score (to a mean of 0.9 out of 1). The study also includes 17 age-matched healthy adults and shows that immediately after stroke and before language ability recovers, patients have a significant increase in the occurrence of a basin state involving only the subcortical and cerebellar areas. The authors conclude that acute poststroke aphasia is associated with “constrained, low dimensional brain dynamics” on the basis of MEM/ELA that coincide with temporary loss of language ability. While the sample size is small, the effect size of stroke is expected to be large, and this study demonstrates a clinically relevant relationship between MEM/ELA features and cognitive function.

####### Alzheimer’s disease:

Fortel et al used a novel function-by-structure embedding framework combined MEM with structural connectivity to estimate excitation-inhibition (E/I) balance and criticality in Alzheimer’s disease risk (Fortel et al., 2022), with structural connectivity derived from diffusion MRI tractography. Applied to 76 cognitively intact middle-aged participants, this model outperformed unconstrained models and detected sex-specific E/I differences in APOE-ε4 carriers, offering a computational approach for early detection of network vulnerability. More specifically, the method revealed that female carriers showing increased E/I ratios toward hyperexcitation and lower critical temperatures suggesting reduced tolerance to network perturbations. These findings align with known vulnerability patterns in Alzheimer’s disease, where female APOE-ε4 carriers face four times higher risk than males.

Another study examined the DMN, the SN, and the CEN among 33 patients with Alzheimer’s disease and 39 controls from the Alzheimer’s Disease Neuroimaging Initiative (ADNI) (Li et al., 2023). The authors fitted the pairwise MEM and used an ELA using a published method(Ezaki et al., 2018). Instead of each’s activity being binarized, the authors binarized the network activity based on whether its activity was above or below its mean. This resulted in 2^³^=8 possible brain states. To simplify the analysis and focus on network dominance, they identified the network with the highest activity at each time point resulting in three “dominant” states: State 1 (DMN dominant), State 2 (CEN dominant), and State 3 (SN dominant). Authors reported that the AD group spent significantly more time in State 1 (DMN dominant) while controls spent more time in State 2 (CEN dominant) and State 3 (SN dominant). The energy barriers for transitioning from State 2 (CEN) to State 1 (DMN) and from State 3 (SN) to State 1 (DMN) were significantly lower in the AD group compared to controls. All three networks showed higher transition frequency through minor states in AD than in controls, but direct transition was lower in AD than in controls. However, within AD patients, direct transition frequency was higher than indirect transitions. Further, AD patients spent more time in minor states than healthy controls. Correlation with cognitive scores showed that the dwell time in DMN dominant state was negatively correlated with MMSE scores and positively correlated with ADAS-Cog scores suggesting that spending more time in the DMN state is associated with worse cognitive performance. The energy barriers, CEN to DMN, and SN to DMN were positively correlated with MMSE scores and negatively correlated with ADAS-Cog scores. This means that lower barriers were associated with worse cognitive performance. Overall, the authors found that the brain dynamics were significantly altered in AD, characterized by a shift toward the DMN-dominant state spending more time in the DMN-dominant state than healthy controls. Since DMN is typically suppressed during goal-directed tasks, and its hyperactivity at rest or insufficient suppression may interfere with cognitive processes suggesting that the AD brain is “stuck” in this internal, self-referential state.

Characterization of Alzheimer’s disease has also been proposed to be enhanced by MEM/ELA approaches (Xing et al., 2024). A recent study examined 30 Alzheimer’s patients and 30 controls. This study relied on resting state data and group ICA to identify functional networks. The MEM and ELA were produced in the typical manner, and then basin dwell times and transition frequencies were compared. Significant correlations between certain state transition frequency and auditory verbal learning were observed. The authors conclude that the energy landscape alterations associated with Alzheimer’s are most likely *compensatory.*

####### Schizophrenia and psychosis risk states:

######## Adult schizophrenia:

Recent MEM/ELA literature has pushed the field towards analyzing the interaction of multiple large-scale brain networks. Because the MEM is constrained to small system sizes, one study takes the strategy of representing the entire DMN as one node, alongside a single-node spatial representation of the visual, somatomotor, dorsal attention, salience, limbic, and FPN (Ishida et al., 2024). While necessarily less granular than studying each network in terms of its constituent parts, such a strategy makes it possible to study the whole brain in the MEM context. Ishida and colleagues examined 36 patients with schizophrenia, 42 patients with major depressive disorder, and 50 healthy controls and found that in schizophrenia the DMN-of and visual-of states had diminished occurrence and duration compared to controls and depression groups. Schizophrenia patients also had increased occurrence of states involving DMN suppression and visual activation. Verbal fluency negatively correlated with the transition from that state to that state’s opposite (*from* DMN-of and visual on *to* DMN-on and visual-of) in schizophrenia. The findings indicate that schizophrenia is a more sever disorder than depression in terms of disruption to brain dynamics.

######## Adolescent-onset schizophrenia (AOS):

Our group examined the energy associated with executive function performance in adolescent onset schizophrenia (Theis et al., 2025). Patients showed more frequent high-energy states, while healthy controls occupied more stable, low-energy configurations. More than 500 independent MEMs of regions associated with executive function in different combinations were ft. A majority of models showed a negative correlation of total energy and executive function performance along with a positive correlation with negative symptom severity. Both groups had two primary basins; controls had deeper basins with larger volumes and higher walls, but they were shallower with lower walls and lower basin volumes among patients. The energy landscape indicated that the trajectory hovered within the high energy domains among patients while controls’ trajectory was in the low energy domain during the task. The significance of these findings is that the network configurations that occur at lower probabilities, i.e. high energy states were associated with schizophrenia while high probability configurations (low energy states) occurred more frequently among healthy adolescents. This suggests that the brain visited certain configurations more often in controls that are possibly more stable configurations that were associated with better performance on executive functions, while low probability configurations that were less stable were abundant in patients. Energy values positively correlated with severity of psychopathology. These findings demonstrate that MEM-derived energy landscapes can reveal meaningful network configurations relevant for understanding brain disorders and may guide the development of circuit-based interventions (Siddiqi et al., 2023; Siddiqi et al., 2024).

######## Clinical High Risk (CHR):

Pairwise MEM applied to resting fMRI data from CHR subjects (n=50), reported that the local minimum brain activity patterns were similar between CHR and controls after examining seven different RSNs (DMN, FPN, SN, DAN, visual, somatomotor, and limbic networks) (Kindler et al., 2024). Using a similar approach as above(Ishida et al., 2024), the activity pattern of each RSN at each timepoint was used to binarize the networks. The states were grouped as main and minor based on the activity patterns of each network, which were identical between the groups. They found that the duration and occurrence of one of the minor states (state 4) representing DMN, limbic, SN, and somatomotor networks being active while others were inactive, was longer and higher in CHR compared to controls. They also found that the transition frequency from state 2 (DMN, limbic, visual, and FPN being active while others were inactive) to 4 and from state 4 to 1 (FPN, SN, DAN, somatomotor, and visual being active while others were inactive) were significantly higher in CHR relative to controls. However, these features were not correlated with the severity of subclinical psychotic symptoms and only showed uncorrected significances with multiple cognitive tasks.

####### MEM applied to understand the effect of interventions:

######## Lysergic acid diethylamide (LSD):

In an interesting examination of the usefulness of perturbing the system using a pharmacological agent (LSD) and examining the complex brain dynamics changes, Ruffini et al (2023) applied statistical physics principles by analyzing three 7-minute resting fMRI data on 15 healthy individuals—specifically, the Ising spin model—and algorithmic complexity measures with an aim to quantify changes in brain criticality and complexity compared to placebo. Only the first and last sessions of resting fMRI data were used in this analysis under each of the LSD and the placebo conditions. The brain was parcellated into 90 AAL regions and the BOLD fMRI data was binarized to fit the MEM. In addition to h and J parameters, the Ising temperature that represents the randomness/disorder in the system were estimated. Data from 15 participants and 4 conditions were concatenated to produce global “archetypes” and condition archetypes by concatenating data from each condition. The authors also examined spin dynamics using Metropolis algorithm, conducted complexity analyses, compared Ising connectivity (J parameter) with structural connectivity using dMRI data, and correlated these measures with subjective experience. The main findings were that LSD increased Ising temperature significantly compared to placebo (mean increase ~6.6%), although both LSD and placebo states were above the critical point (paramagnetic phase), LSD shifted further away from criticality, and that the homotopic connectivity (interhemispheric links between symmetric regions) was reduced under LSD, and contributing to increased disorder. The complexity measure using the block decomposition method correlated with Ising temperature with a medium effect size. Ising connectivity correlated with dMRI-derived structural connectivity and LSD was observed to reduce homotopic functional connectivity, especially in regions like hippocampus and thalamus. However, the correlations between questionnaire scores (e.g. imagery, emotional arousal) and Ising temperature or complexity metrics were weak. This study is similar in approach to the study that examined rTMS induced changes in ELA (Fan et al., 2024) (discussed later).

######## Repetitive transcranial magnetic stimulation (rTMS):

Fan et al (2024) reported on changes in energy landscape before and after 1 Hz rTMS on two distinct regions: the left frontal lobe and the left occipital lobe. This paper represents a significant and methodologically sophisticated contribution to the field of noninvasive brain stimulation by successfully using pairwise MEM to move beyond conventional static FC analysis to characterize the complex, dynamic, and global effects of rTMS on large-scale RSNs. This dynamic perspective helps understand how a local perturbation propagates through the brain as a complex dynamical system. The study clearly and compellingly demonstrates that the same stimulation protocol yields significantly different, target-dependent dynamic responses that demonstrates the importance of stimulation site hierarchy. Occipital lobe rTMS resulted in decreased state transitions suggesting a less flexible or more stable dynamic states, while frontal lobe rTMS induced different changes in less-dominant states. The analysis goes deeper than this by quantifying state duration, transition frequency, and the minimum energy cost (shortest path) between states. The shortest path analysis revealed that the minimum energy cost for functional state transitions was lowest after occipital lobe stimulation and highest after frontal lobe stimulation providing a powerful new way to quantify the ease or difficulty of switching between functional brain states after intervention. However, the study was conducted on a small sample (n=15), and the methodology required concatenating time series data across individuals. While necessary for model stability, this approach inherently neglects individual differences, which are critical for the clinical application of rTMS. The study focused exclusively on the effects of 1 Hz inhibitory rTMS. Extending these findings to other protocols (e.g., high-frequency, TBS) and brain sites would help in creating a comprehensive “stimulation map.” This study supports that the energy landscape framework offers a vital perspective for understanding how local brain stimulation globally restructures brain activity, which is essential for guiding and optimizing future clinical rTMS applications.

This set of studies viewed collectively, suggest that psychopathology can be conceptualized along a spectrum of energy landscape dynamics. On one end lies pathological rigidity, characterized by overly deep basins and high energy barriers that trap the brain in repetitive states. This phenotype is evident in autism spectrum disorder (overly stable intermediate states), aging (reduced transition fluidity), and depression (rumination in DMN attractors). On the opposite end lies pathological instability, where energy landscapes are flattened, barriers are eroded, and the brain transitions chaotic without sustaining stable representations. This phenotype characterizes Schizophrenia and psychedelic states induced by LSD. This “Rigidity-Instability” framework moves beyond symptom checklists to offer a mechanistic taxonomy of brain disorders based on dynamical systems theory.

## 5. How MEM Advances Beyond Traditional fMRI Analysis Approaches

The application of the pairwise MEM to fMRI data offers several significant advantages that could considerably advance neuroimaging research beyond widely used fMRI analyses to identify differences in regional BOLD signal intensity and in features of sFC and dFC. Table 2 compares and contrasts sFC and dFC with MEM.

The BOLD signal is rich in information on brain activity. The traditional fMRI approaches take advantage of only a small portion of this signal while a large proportion is either smoothed or averaged out. More specifically, temporal averaging smooths out dynamics of brain activity within the timescale of observation in first-order models. The MEM provides a paradigm shift and a unique mechanistic framework for understanding brain states. The energy landscape derived from MEM represents a global structure of probability distribution of all observed network configurations, offering a theoretical bridge between observed neural activity and underlying physical principles since it better reflects neurophysiological principles of brain activity underlying cognitive performance and psychopathology, which may be a better indicator of brain activity dynamics than higher/lower activation or hypo/hyperconnectivity measured in traditional analytical approaches. This is because a time-resolved pattern of activation and deactivation of regions in a network better represents the neural dynamics than a few activated regions or correlated region pairs. In addition, the functional interactions estimated by MEM indexes structural connectivity (Watanabe et al., 2013) and gray matter (Watanabe et al., 2014a) more accurately than the correlation-based FC reflecting the constraint on brain function placed by anatomical structure and connections. Thus, the MEM models are considered closer to the ground truth of brain network that can be leveraged to develop biophysically informed models to link energy state transitions to neurotransmitter dynamics, as suggested by integrating neural mass models with energy landscape (Deco and Jirsa, 2012). This approach could map how pharmacological interventions alter the energy landscape predicting treatment responses in psychiatric disorders (Braun and Mattia, 2010; Braun et al., 2021). Furthermore, characterizing neurobiology as alterations in energy barriers between functional states provides a more nuanced perspective on brain dysfunction (Gu et al., 2018; Watanabe et al., 2014b). Although energy barriers are mathematically derived theoretical constructs, they index the “difficulty” in switching from one pattern of network configuration to another suggesting altered flexibility of networks in adapting to changing cognitive demands.

MEM overcomes a major limitation of current methods such as sFC and dFC. MEM accounts for correlated regional activations in static FC thus, overcoming a major confound. The dFC and MEM are the two prominent approaches that attempt to capture changes in distributed neural activity in to decode the complexities of large-scale functional networks. The dFC approaches rely on arbitrarily defined temporal windows by modeling interactions across temporal scales simultaneously. Newer methods such as change point detection applied to dFC does not need pre-set window sizes, but it relies on statistically significant shifts in network architecture that may span multiple TRs requiring temporal averaging which assumes that the pairwise correlations among regions are independent (Hindriks et al., 2016; Lindquist et al., 2014). While the dFC emphasized time-resolved patterns of co-activations, MEM captures the statistical structure of evolving brain states under specific constraints in addition to time-resolved coactivation patterns (Table 2). Further, the MEM framework captures emergent network properties at the timescale of each TR while offering a more comprehensive view of brain dynamics than dFC (Ezaki et al., 2017). MEM parameters (*h* and *J*) may serve as more sensitive and specific biomarkers for neuropsychiatric disorders than conventional FC measures. First-order parameters, h, can detect regional dysfunctions that correlation-based sFC and dFC may miss, while second-order parameters, J, estimate direct interactions, helping to distinguish primary from secondary network abnormalities. This approach has already shown greater sensitivity in differentiating schizophrenia patients from healthy controls(Yang et al., 2014). Besides, modeling the full probability distribution of brain states can reveal subtle shifts in state occupancy that may predict disease progression or treatment response, extending beyond what mean activation or connectivity strength can offer (Ezaki et al., 2017; Olsen et al., 2024).

Unlike FC, which conflates direct and indirect associations, the MEM interaction parameter (J) isolates the direct statistical coupling between regions by factoring out the influence of the rest of the network. While pairwise MEMs are undirected and thus do not infer temporal causality in the Granger sense, their generative nature allows for in silico perturbation. This enables researchers to simulate how altering specific edges would reshape the global energy landscape, offering a predictive framework like structural control theory (Gu et al., 2018). This could inform intervention models that identify optimal neuromodulation targets, supporting advances in personalized brain stimulation (Shine et al., 2019). Furthermore, integrating MEM with computational causality frameworks like dynamic causal modeling (DCM) (Friston et al., 2003) or Granger causality could help distinguish direct from indirect influences on brain networks, addressing a key limitation of conventional FC methods.

Task-based fMRI studies could benefit greatly from MEM approaches by characterizing task performance as trajectories through an energy landscape rather than just activation patterns. Watanabe et al (2014c) demonstrated this in bistable perception, revealing how visual task performance relates to specific transitions between energy states. Similarly, our work showed that trajectory of energy states in adolescents with schizophrenia remained primarily in high-energy states during an executive function task, while controls stayed in lower-energy domains (Theis et al., 2025) suggesting that while controls relied on more stable network configurations throughout the task execution, patients relied on unstable network configurations. Identifying task-specific attractor states and the energy barriers involved in cognitive processing offers new insights into the neural cost of cognitive operations. Additionally, modeling individual differences in cognitive strategy as variations in energy landscape topology could explain why some individuals struggle with certain tasks despite similar activation patterns, extending Braun and Mattia’s work(Braun and Mattia, 2010) on attractor dynamics in decision-making. Despite these major advantages of MEM, it is often misconstrued that dFC provides the same information as MEM does.

Simultaneous EEG-fMRI data could be integrated by modeling fast electrophysiological dynamics as transitions between energy states identified through fMRI using multi-scale neural dynamics(Deco and Jirsa, 2012). Structural connectivity from diffusion imaging could inform priors on the J parameters in the model to better understand the structure-function relationships (Gu et al., 2018; Watanabe et al., 2013). Additionally, integrating with PET imaging for neurotransmitters may help explain variations in energy barriers between brain states linking molecular mechanisms to network dynamics(Shine et al., 2019). However, there are limitations in integrating PET studies with MEM applied to fMRI because of poor spatial resolution of PET, and PET cannot provide data on temporal fluctuations of neurotransmitters. Further, PET studies provide one aspect of neurotransmitter such as density of selected receptors. The latter could be a major problem in certain complex systems such as glutamate which has a variety of receptors that could be an interpretational challenge.

## 6. Future directions to address limitations

### 6.1 Scalability with Computational advances

While pairwise MEMs can model the activity of smaller neuronal ensembles with near-perfect accuracy, it becomes less accurate when larger populations and different neuronal types are considered together since pairwise correlations alone often fail to capture the full dynamics of larger networks; however, including higher-order couplings can overcome this limitation (Olsen et al., 2024; Zanoci et al., 2019). In addition, the exponential growth of state space ($2^{N}$) with increasing number of neurons (N) will require dimensionality reduction or sparse sampling methods (Tkacik et al., 2014). Alternatively, certain networks can be treated as a “node” that could allow for modeling larger neural systems (Ishida et al., 2024; Kindler et al., 2024) followed by post hoc analysis of each network. However, such an approach may lose granularity of nodal activity and connectivity in each network when modeled together. The MEM needs to include higher number of regions to expand the usefulness of application of MEM to larger networks. Further development of the MEM methodology to address this limitation is warranted.

Adding number of timepoints in addition to the number of regions adds to this challenge. A 9-neuron system with 3 time bins (T) would yield approximately ($2^{NT}$) 135 million states in a 9-neuron system(Yeh et al., 2010) making computation highly demanding. With standard fMRI TRs (e.g., 1 second), acquiring data to cover all possible states would take thousands of hours. Although these numbers reflect the number of possible states, the number of observed states may be fewer than the possible states. Developing temporal MEM variants that explicitly model state transition dynamics over multiple timepoints rather than treating time points independently could better capture sequential cognitive processes even better. Future advances in computational methods, efficient estimation algorithms, and more efficient data acquisition strategies will be crucial for scaling MEMs to whole-brain networks.

Despite some of these challenges, application of dynamical systems perspective to investigate neural mechanisms underlying psychiatric disorders is growing (Kheirkhah et al., 2025; Loh et al., 2007). However, it is necessary to be able to causally link the insights gained from the application of dynamical systems tools to clinical manifestations to move the field toward designing novel treatments as well as providing a neurobiological basis for clinical diagnosis (Lewis et al., 2025c).

### 6.2 Methodological Limitations and needed improvements

The need to binarize the activation patterns into “on” or “of” to the continuous BOLD signals is often criticized. Prior studies show that MEM, based on the binarized BOLD signal, better reflected the underlying structural connectivity, the ground truth of brain networks than traditional FC method that rely on continuous BOLD signals (Watanabe et al., 2013). The same study also showed that ternary quantization instead of binary did not improve results of MEM-based analysis.

Since fMRI’s spatial resolution at the voxel level reflects activity of thousands of neurons (Drew, 2019), fMRI-based MEMs simplify heterogeneous neural populations into aggregated on/of regional states creating a mismatch with the cellular-level theory and voxel-level measurements. However, modeling BOLD data at the voxel/regional level can reveal population-level dynamics that may be more relevant for linking energy states to cognition and behavior. Many studies have employed network level dynamics to understand the interaction between the networks (Ishida et al., 2024; Kindler et al., 2024). Neurovascular coupling variability across brain regions (Iadecola, 2017; Schaefer and Iadecola, 2021) can complicate interpretation, as excitatory and inhibitory neurons produce different BOLD response patterns (Moon et al., 2021). The temporal lag between neural firing and hemodynamic responses may not align with the instantaneous binary states assumed by MEMs (Logothetis, 2007) because binarizing continuous BOLD signals needs consensus that can be addressed with principled approach to binarizing the BOLD signals. Additionally, the equilibrium assumptions underlying the Ising model may not adequately capture the non-equilibrium nature of neural systems, where interactions are directional and asymmetric. Ongoing development of non-equilibrium statistical mechanics approaches, such as constrained maximization of Shannon entropy, may better address these limitations (Dewar, 2005).

Systematic investigation of when higher-order terms beyond pairwise interactions are necessary to improve model accuracy for complex network dynamics (Schneidman et al., 2006). Combining MEM with EEG or MEG for faster electrophysiological dynamics, structural connectivity from diffusion imaging to inform J parameters, and PET imaging for neurotransmitter function could provide a more comprehensive brain mapping (Deco and Jirsa, 2012; Shine et al., 2019; Watanabe et al., 2013). Integrating MEMs with biophysically realistic models could bridge statistical descriptions with mechanistic understanding, potentially linking energy state transitions to neurotransmitter dynamics and predicting treatment responses (Shine et al., 2019).

### 6.3 Clinical Translation and Generalizability

Biological interpretation of MEMs can be affected by sampling biases, especially when the actual entropy distribution falls outside the model class, as can happen with a 2^nd^-order Ising model(Macke et al., 2011), particularly in large cell populations. Using a generalized Ising model that includes both 1^st^- and 2^nd^-order terms helps reduce this risk. Current MEM applications focus primarily on homogeneous samples, limiting generalizability across diverse populations. Future studies should examine developmental changes, age-related alterations in neurovascular coupling, and disease-specific pathophysiology that may significantly reshape energy landscapes(Calabro et al., 2024). The computational intensity and specialized expertise required for MEM implementation currently limit clinical adoption. Developing robust pipelines with normative databases, standardized protocols, and validated clinical thresholds will be essential for widespread application.

### 6.4 Clinical Applications and Biomarker Development:

MEM parameters (h and J values) offer potentially superior biomarkers compared to conventional FC measures. First-order parameters can identify regional dysfunctions missed by correlation-based analyses, while second-order parameters estimate direct interactions, distinguishing primary from secondary network abnormalities (Yang et al., 2014). The full probability distribution of brain states could reveal more refined alterations in state occupancy that predict disease progression or treatment response (Ezaki et al., 2017). Task-based applications show particular promise, as demonstrated in studies of bistable perception (Watanabe et al., 2014c) and executive function in schizophrenia (Theis et al., 2025). Characterizing task performance as trajectories through energy landscapes rather than simple activation patterns offers novel insights into the neural cost of cognitive operations.

Beyond serving as static biomarkers, MEMs offer a computational sandbox for predictive psychiatry. Because the MEM is a generative model, it can serve as a patient-specific “Digital Twin.” Researchers can mathematically perturb parameters—simulating the “lesioning” of a node or the “stimulation” of an edge—to predict how the global energy landscape would reconfigure before an intervention is applied. This capability is particularly relevant for guiding neuromodulation (TMS/DBS). By mapping a patient’s specific energy barriers, clinicians could theoretically identify the optimal stimulation target required to “flatten” a pathological basin (e.g., in depression) or “deepen” a stable attractor (e.g., in schizophrenia), transforming MEM from a descriptive tool into a prescriptive guide for precision medicine

### 6.5 Paradigm Shift and Broader Implications:

The application of MEM to fMRI represents a fundamental shift from descriptive to mechanistic models of brain function. By grounding analysis in statistical physics principles, MEM provides a theoretical framework that can potentially unify disparate neuroimaging observations and bridge microscale neural activity with macroscale brain function (Rabinovich et al., 2008). This approach enables characterization of neuropathology as alterations in energy barriers between functional states rather than simply connectivity differences, offering a more nuanced perspective on brain dysfunction. As computational methods advance and these limitations are systematically addressed, the MEM approach is poised to transform functional neuroimaging analysis, ultimately leading to deeper insights into both normal brain function and the pathophysiology of neuropsychiatric disorders.

## Figures and Tables

**Table 1:** Peer-Reviewed Publications Utilizing Maximum Entropy Models on fMRI Data

Publication (First Author, Year)	Sample size	Task/resting	Key Finding / Significance	Comments
**Core Methods & foundational applications**				
Kim et al (2000)	*3 datasets; each with 60 whole brain acquisitions at 3 secs apart for motor, auditory, and visual cortical stimulation*	*Dominant hand movement (motor); verb generation (auditory), and alternating checkerboard (visual)*	Mutual information-based nonparametric method that included Ising prior performed superior by reducing spurious ‘activations’ in neighboring voxels	Full details of fMRI data and the sample size not provided
Fraiman et al (2009)	*5 healthy females*	*Resting state fMRI*	Demonstrated that fMRI networks are statistically indistinguishable from the Ising model near its critical temperature.	Set out to examine whether there is a dynamical equivalent of resting state
Risser et al (2009)	*An fMRI data to map sensory cortices (auditory, motor, and visual) and during reading and computation*	*Tasks as stated*	To estimate how much the neighboring voxel’s activation is, temperature parameter is used. The paper attempts to find the perfect temperature to 500 parcels and finds that the one with unsupervised setting performed the best.	Application on the real fMRI data was based on simulation experiment
Watanabe et al (2013)	*6 healthy adults*	*Resting*	Coefficients reflected anatomical connections more accurately than traditional FC, establishing pMEM as a superior connectivity measure.	Foundational rs-fMRI MEM paper; long single-subject scans improved parameter estimation; validates pairwise sufficiency for describing RSN activity
Watanabe et al (2014a)	*6 healthy adults*	*Resting state fMRI state fMRI*	ELA mapped the energy landscape during bistable perceptions assigning stable perceptions to one system	Method expansion from MEM fts to landscape analysis (disconnectivity graphs, MCMC) to map state transitions
Kang, Pae & Park (2017)	*Subcortical network dataset (see paper)*	*Resting state fMRI*	Subcortical system exhibits multistability with hub-like transition states; baseline network yields longer paths vs. altered coupling.	Applied energy landscape to subcortical ROI set; emphasized transition-state hubs and coupling manipulations. Large sample size (n=470), and rigorous mathematical framework
Ezaki et al (2017)	*2 healthy controls from HCP data*	*Resting state fMRI*	Thress systems were examined (DMN, FPN, and CON). MEM-ELA on fMRI shows **age-related** changes in ease of dynamical transitions (DMN/CON)	Extended MEM/landscape to aging; compared energy barriers and basin structures between age groups.
Ashourvan et al (2017)	*20 healthy participants*	*Resting state fMRI*	Examined how functional modules dynamically interact with one another for cognitive performance. Results showed three classes of regions with similar patterns of allegiance to community states. Visual, attention, sensorimotor, and subcortical ROIs formed one functional community while the remaining regions formed executive control or default mode and salience community	Functional brain states were defined based on allegiance of regions to dynamic functional communities. MEM was applied to functional connectivity profiles as opposed to functional activation profiles as done by other studies
Kang et al (2019)	*HCP Young Adult dataset (see paper)*	*Resting state fMRI*	Graph-theoretic analysis of MEM-derived state-transition networks from HCP rs-fMRI.	Graph-theoretic analysis of transition networks built on MEM-derived landscapes; open data; large sample
Ruffini et al (2023)	*15 healthy participants*	*Resting state fMRI*	individualized critical temperatures for phase transition were higher in the LSD condition than in the placebo condition	Relatively short fMRI data. Advances the concept of pharmacological
**Estimation advances**				
Kang et al., (2021)	*468 participants from the HCP database*	*Resting state fMRI*	Bayesian MEM (BMEM) supports subject- specific landscapes with better small-sample properties	Variational Bayes approach; demonstrates subject specificity and links to behavior
Jeong et al. (2021)	*298 children (205 males and 93 females; 233 ADHD children and 65 control children*	*Resting state fMRI*	Confirmed superior sensitivity of pMEM parameters over correlation in clinical group analysis (ADHD).	Nonlinear dynamic properties observed on the pairwise MEM differ for each group, and pairwise MEM parameters were shown to be more sensitive to group differences and better associated with the behavior scores of ADHD compared to the Pearson correlation-based functional connectivity
Khanra et al (2024)	*Midnight scan club; 10 healthy subjects for 10 consecutive nights for 5 hours*	*Resting state fMRI*	Within-subject test–retest reliability exceeds between-subject; variational Bayes individual-level MEM comparable to MLE.	Reliability/validation study; proposes permutation test for landscape indices; practical guidance for individual ELA.
Hosaka et al (2025)	*HCP YA data S1200 release, 1002 subjects*	*Resting state fMRI*	Energy landscape shape and first-order transition probabilities are highly similar between real and surrogate data. ELA’s first-order structure can be explained by stationary, linear properties rather than genuine switching among nonlinear attractors	Focuses on first-order output only and underplay higher order interactions
Gu et al (2018)	*48 healthy subjects for diffusion spectrum imaging, resting fMRI on 25 subjects*	*Resting state fMRI*	Maximum Entropy Model Structure-Function Integration	Used MEM informed by diffusion imaging to quantify the energy landscape, validating predictions against rsfMRI activation rates.
**Clinical applications**				
Watanabe et al (2014b)	*14 healthy subjects*	*Sleep fMRI, EEG, EMG, and eye movement data*	Basal brain activity and connection strength increased in the DMN during slow wave sleep and decreased during REM periods; network activity was in opposite direction in the FPN and SMN	Did not differentiate between the early and late stages of each sleep phase separately Examined within sleep stage modulation of brain activity
Watanabe & Rees (2017)	*24 high functioning adults with autism compared to 26 typically developing adults*	*Resting state fMRI*	Neurotypical brains frequently transitioned between two major states via an intermediate state; ASD brains showed fewer indirect transitions because the intermediate state was unstable, and this reduction predicted ASD symptom severity	Elegant, mechanistic link from MEM-derived dynamics to behavior. Stationary pMEM + random-walk simulation may miss nonstationary/higher-order effects.
Ezaki et al (2018)	*28 young and 28 older healthy adults*	*Resting state fMRI*	Applied ELA and reported that the major activity patterns (all active and all inactive) were similar between the groups, the transitions between these two states were less frequent in older relative to younger adults. This correlated with “efficiency” that was more prominent in the DMN than in the cingulo-opercular network.	Cross-sectional study on different groups of individuals; use of resting fMRI data; small samples
Fan et al (2022)	*16 post-stroke aphasia patients (15 analyzed) + 17 controls*	*Task (Auditory sentence comprehension)*	Acute/subacute aphasia shows restricted dynamics with two dominant states; flexibility increases by chronic stage; dynamics relate to language scores recovery	Energy landscape across recovery stages; MEM on network-averaged signals; longitudinal design highlights reorganization
Fortel et al (2022)	*76 cognitively intact middle-aged persons*	*Hybrid resting state- functional connectome*	Structural network constrained functional interactions were better than the unconstrained model, thereby improving that pairwise MEM. This novel approach showed sex differences among APOE-ε4 carriers in functional dynamics at criticality that female carriers exhibited a lower tolerance to network disruptions due to higher excitatory interactions	Small sample of healthy individuals for risk of AD.
Li et al (2023)	*33 Alzheimer’s disease patients and 39 healthy controls*	*Resting state fMRI*	Triple-network (DMN–FPN–SAL) landscapes differentiate groups; disease progression reflected in basin structure and transition barriers.	Focus on triple-network ELA; emphasizes diagnostic potential of landscape metrics.
Kindler et al (2024)	*50 clinical high-risk (CHR) + 33 controls*	*Resting state fMRI*	CHR group shows altered dwell time and transition ease among DMN/salience/frontoparietal states.	MEM-based ELA at risk state; supports prognostic utility of landscape dynamics.
Ishida et al (2024)	*50 HC, 36 schizophrenia, 42 MDD*	*Resting state fMRI*	SZ shows more disrupted dynamics than MDD; altered duration/occurrence of large-scale network states; links to verbal fluency in SZ.	MEM-based landscape over seven canonical networks; compares psychiatric groups to controls.
Xing et al (2024)	*30 Alzheimer’s disease, 30 controls (ADNI)*	*Resting state fMRI*	Three stable states identified; AD shows reduced transitions and altered state occupancy compared to controls.	Applies ELA to AD; interprets differences in attractor topology and switching ease.
Fan et al (2024)	*23 healthy individuals*	*Resting state fMRI*	Examined 7 RSNs before and after 20 min of 1Hz rTMS at 100% threshold to the left frontal and occipital lobes.	Small sample size
Theis et al (2025)	*26 adolescent onset schizophrenia and 46 controls*	*Penn Computerized Exclusion Test task for executive function*	Examined a network of regions associated with executive function finding higher energy in adolescent onset schizophrenia that was correlated positively with severity of psychopathology and negatively with executive function performance	Relatively small sample size

**Table 2:** Comparison of static and dynamic FC with MEM modeling

	Static FC	Dynamic FC	MEM modeling
**Theoretical foundations**	Based on pairwise correlations or other dependencies of BOLD signals between regions with the assumption of stationarity and highly correlated regions being functionally connected	Grounded in the notion that FC is inherently time-varying identifying patterns of FC changes over time	Although MEM is a form of dFC, it is rooted in statistical physics & information theory, particularly the principle of maximum entropy
**Unique identified features**	Average correlations reflecting synchronized activations between pairs of regions	Focuses on how networks reconfigure revealing transient nature of networks that may index in cognitive/behavioral context	Captures global structure of brain activity by modeling joint probability of regional activation states with pairwise correlations as constraints
**Independence of pairwise correlations**	Independence of pairwise correlations are assumed	Independence of pairwise correlations are assumed	Non-independence or interdependence of coactivations are explicitly acknowledged
**Modeling basis**	2^nd^-order only	2^nd^-order only	Both 1^st^- & 2^nd^-order
**Temporal resolution**	Poor (averaged over minutes); Entire duration of data acquisition or task block	Moderate, resolves functional states on the order of seconds to minutes, depending on window size and methodology	Each TR (~0.5–2 sec); MEMs are typically static models applied to temporally aggregated data inferring structure from the co-occurrence of states and do not natively model transitions over time
**Temporal dynamics**	Does not reflect temporal changes in network architecture	dFC is explicitly designed to examine temporal fluctuations in FC that can detect dynamic changes associated with cognitive events, arousal, or disease states	Energy landscape from MEMs interpreted as dynamic state transitions & conceptualized as movement across the landscape when dynamical systems applied
**Evolution of the networks**	Not explicitly modeled since the network is based on temporal averaging	Demonstrates shifts in network architecture when such a shift is statistically significant (CPD) and dwell time, transitions, and switching rates as in sliding window	Energy landscape and examination of trajectory can identify evolution of the brain network toward the local minima that correspond to recurrent network states (attractors)
**Structural constraints as ground truth**	Less reflective of ground truth structural constraints	Less reflective of structural constraints as ground truth	Better reflective of structural constraints such as gray matter and structural connectivity
**Trajectory of BOLD signal variations**	Not modeled	Not modeled	Modeled for each TR that can be linked to the stimulus and response
**Effect Sizes (Cohen’s *d*)**	Moderate (0.3–0.8 for group differences)	Variable (0.2–1.2 depending on window) (Zhang et al., 2018)	Large (0.8–1.5 for patient vs control discrimination)(Theis et al., 2025)
**Test-retest Reliability (ICC)**	Good (0.6–0.8)	Poor to Moderate (0.2–0.6) (Zhang et al., 2018)	Good to Excellent (0.7–0.9)(Watanabe et al., 2013)
**Computational Complexity**	Low (O(n²))	Moderate (O(n^²^×w), w=windows); dFC scales reasonably well with current computational resources and are widely applicable across whole-brain data	High (O(2^ⁿ^), but tractable for n<20); Modeling higher-order dependencies are challenging due to exponential relationship of energy states with the number of regions
**Biological plausibility and neural mechanisms**	Correlation-based with relatively poorer concordance with structural connectivity; causal connectivity may be much sparser(Lewis et al., 2025a)	dFC resonates with the non-stationary and adaptive nature of the brain, and captures shifts between functional states and mirrors the electrophysiological recordings and behavioral state transitions	Offer a mechanistic view rooted in thermodynamic systems, modeling the probability of brain states given constraints. Attractor dynamics and energy minima has parallels in neural population models and memory encoding theories
**Relationship with disease and behavior**	Low to Moderate	Linked to cognitive processes such as attention, memory, or task engagement; likely better with CPD (Anastasiou et al., 2022; Barber et al., 2018; Kundu et al., 2021; Zheng et al., 2022)	High (captures state occupancy differences) (Theis et al., 2025; Yang et al., 2014); Energy states can align with cognitive functions or disease traits, particularly when attractor dynamics are considered
**Biological interpretability**	Graph theory principles are often used	Limited but better than sFC due to sensitivity to temporal fluctuations (correlation-based); Identifies recurrent network states or “brain states” whose dwell times, transitions, and switching rates	Energy framework(Watanabe et al., 2013) offers interpretable statistical structure of energy associated with specific network configurations. Low-energy (high-probability) states may reflect preferred network configurations
**Relationship with cognition and behavior**	Correlation with behavioral and cognitive measures are reported	May be directly linked to cognitive processes such as attention, memory, or task engagement	Energy states can align with cognitive functions or disease traits when attractor dynamics are considered
